# m6A Topological Transition Coupled to Developmental Regulation of Gene Expression During Mammalian Tissue Development

**DOI:** 10.3389/fcell.2022.916423

**Published:** 2022-07-05

**Authors:** Shanshan Li, Qing Yang, Rui Jiao, Pengfei Xu, Yazhou Sun, Xin Li

**Affiliations:** ^1^ School of Medicine, Shenzhen Campus of Sun Yat-sen University, Shenzhen, China; ^2^ The Seventh Affiliated Hospital of Sun Yat-sen University, Shenzhen, China; ^3^ Guangdong Provincial Key Laboratory of Digestive Cancer Research, The Seventh Affiliated Hospital of Sun Yat-sen University, Shenzhen, China

**Keywords:** m6A, topological structure, topological transitions, tissue development, gene expression

## Abstract

N6-methyladenosine (m6A) is the most prevalent internal modification and reversible epitranscriptomic mark in messenger RNAs (mRNAs) and plays essential roles in a variety of biological processes. However, the dynamic distribution patterns of m6A and their significance during mammalian tissue development are poorly understood. Here, we found that based on m6A distribution patterns, protein-coding genes were classified into five groups with significantly distinct biological features and functions. Strikingly, comparison of the m6A methylomes of multiple mammalian tissues between fetal and adult stages revealed dynamic m6A topological transition during mammalian tissue development, and identified large numbers of genes with significant m6A loss in 5′UTRs or m6A gain around stop codons. The genes with m6A loss in 5′UTRs were highly enriched in developmental stage-specific genes, and their m6A topological transitions were strongly associated with gene expression regulation during tissue development. The genes with m6A gain around the stop codons were associated with tissue-specific functions. Our findings revealed the existence of different m6A topologies among protein-coding genes that were associated with distinct characteristics. More importantly, these genes with m6A topological transitions were crucial for tissue development *via* regulation of gene expression, suggesting the importance of dynamic m6A topological transitions during mammalian tissue development.

## Introduction

N6-methyladenosine (m6A) is the most prevalent and conserved internal modification in mammalian messenger RNAs (mRNAs) (Motorin and Helm 2011; Jia, Fu, and He 2013; Liu et al., 2014), and it is mainly enriched in the consensus RR (m6A)CH sequence, where R denotes A or G and H denotes A, C or U (Du et al., 2016). m6A modification is widely acknowledged to be a reversible modification that is catalyzed by multicomponent complexes, mainly including “writers” such as methyltransferase-like 3 (*METTL3*) and methyltransferase-like 14 (*METTL14*) (Wu et al., 2016), and demethylated by “erasers” such as obesity-associated protein (*FTO*) and alkB homolog 5 (*ALKBH5*) (Jia et al., 2011; Zheng et al., 2013). In addition, several binding proteins have been reported as “readers,” including YT521-B homology (YTH) domain-containing proteins and the heterogeneous nuclear ribonucleoprotein (HNRNP) family, that recognize m6A modifications on mRNA (Li et al., 2017; Liu et al., 2017). These interactions further participate in regulating mRNA fate in different aspects, including structure (Liu et al., 2015), maturation (Huang and Yin 2018), stability (Wang, et al., 2014a), splicing (Adhikari et al., 2016), translation (Slobodin et al., 2017) and decay (Liu and Zhang 2018). Given the diverse and flexible regulation of gene expression, m6A modification plays important roles in diverse biological processes, such as cancer progression (Cui et al., 2017; Ianniello, Paiardini, and Fatica 2019; Chen and Wong 2020; Liu et al., 2020; Yang et al., 2020; Xu et al., 2021) and embryonic development (Zhang M. et al., 2020), by modulating mRNA metabolism and translation (He et al., 2019).

Numerous studies have revealed that m6A modification is a critical posttranscriptional regulator of gene expression, therefore, the correct deposition of m6A is required for normal development (Frye et al., 2018). Previous studies have described the global landscape of m6A in human fetal and adult tissues and observed significant differences in fetal and adult stages (Xiao et al., 2019; Zhang, et al., 2020a). Moreover, compelling evidence indicates that m6A plays an essential role during mammalian development. It has been reported that genetic knockout of the m6A writer enzyme *METTL3* or *METTL14* results in embryonic lethality in mice (Batista et al., 2014; Geula et al., 2015). Conditional knockout of the m6A reader protein *YTHDF2* in mice also causes lethality, with embryos failing to thrive at late embryonic developmental stages (Li et al., 2018). In addition, m6A modification participates in embryonic and adult stem cell differentiation by regulating transcriptome switching (Roundtree et al., 2017). However, there is limited information regarding the dynamic change in m6A topology during mammalian tissue development and the underlying mechanisms.

Recent studies have revealed that m6A deposited in different genic regions of mRNAs has distinct biological functions. For example, m6A residues in the 5′UTRs have been shown to enhance the translation of mRNAs (Meyer et al., 2015), and m6A enriched in long internal exons participate in pre-mRNA splicing (Dominissini et al., 2012). In addition, m6A modification in the 3′UTRs is significantly associated with alternative polyadenylation (APA), and m6A near the stop codon also affects APA and translation efficiency (Dominissini et al., 2012; Yue et al., 2018). Moreover, a previous study in *Arabidopsis thaliana* reported that mRNAs were endowed with two different types of m6A topology according to the m6A pattern distributed along the transcript (Wan et al., 2015). Although these studies provided insight into how distinct m6A topologies could confer position-dependent functions to mRNAs through diverse molecular mechanisms, little is known about genome-wide m6A topological patterns and their potential functions and how topological transitions regulate gene expression during diverse biological processes.

Here, by integrative analysis of published RNA m6A methylation profiles from multiple tissues of different developmental stages in mammals, we found that all the protein-coding genes can be classified into five groups according to their m6A topologies, and genes with different m6A topologies showed unique biological functions and characteristics. Strikingly, comparative analysis of m6A methylomes between fetal and adult tissues revealed dramatic m6A topological transitions during mammalian development. We identified large numbers of genes with a significant m6A loss in 5′UTRs or gain near stop codons, which were crucial for tissue development. In addition, we also observed similar m6A topological transition patterns in cancer progression. Taken together, our study systematically investigated genome-wide m6A topological patterns across multiple mammalian tissues and developmental stages and suggested a role of m6A topological transitions in the regulation of gene expression during mammalian tissue development.

## Materials and Methods

### Sequencing Data Sources

All the raw fastq data used in this study were downloaded from public databases, including Gene Expression Omnibus (GEO), and Genome Sequence Archive (GSA) databases. The MeRIP-seq and Input RNA-seq data of human adult tissues were collected from GSE122744 (Zhang, et al., 2020a) and CRA001315 (Liu et al., 2020), fetal tissues were collected from GSE114150 (Xiao et al., 2019) and GSE99017 (Yoon et al., 2017). The raw MeRIP-seq and input RNA-seq data of mouse adult tissues were collected from CRA001962 (Liu et al., 2020), fetal tissue were downloaded from GSE99017 (Yoon et al., 2017). The raw MeRIP-seq and Input RNA-seq data of hESC were collected from GSE52600 (Batista et al., 2014). *YTHDF2*’s raw RIP -seq and Input RNA-seq data were collected from GSE142827 (Fang et al., 2021). The ovary cancer’s raw MeRIP-seq and Input RNA-seq data were collected from GSE119168 (Zhang et al., 2019), invasive malignant pleomorphic adenoma’s (IMPA) raw MeRIP-seq and Input RNA-seq data were collected from GSE161879 (Han et al., 2021).

### Sequencing Data Processing

The raw fastq reads in the process of library construction were paired end, and only reads 1 was used for m6A peak identification in the study. First, FastQC (version 0.11.8) was used to access the base quality of raw data, and the raw data that did not pass the criteria of FastQC were filtered. Then we trimmed the adaptor and low-quality reads using fastp (version 0.20.0) with default parameters and checked the data quality again by FastQC. Next, clean reads were mapped to the reference genome assembled by using the hisat2 software (version 2.1.0) and all parameters were set to the default settings. Unmapped and low-quality reads were filtered by samtools (version 1.9) with the ‘−F 0 × 4 −q 30’ parameter. For human data, the hg19 RefSeq gene index was selected and downloaded from hisat2 official website (http://daehwankimlab.github.io/hisat2/download/). For mouse data, we used mm10 as the reference mouse genome and the mm10 RefSeq gene index was also downloaded from hisat2 official website.

To measure the m6A and gene expression level, we firstly acquired the exon information from GTF (hg19, mm10) file, which was downloaded from GENCODE database (https://www.gencodegenes.org/). Then, the reads mapped to exon were counted using FeatureCounts (version 1.6.0) as well as bedtools (version 2.29.0) was used to count the reads mapping to 3′UTRs, 5′UTRs, and CDS, respectively. In general, the counts in m6A-seq (IP) and RNA-seq (Input) were normalized by TPM, which is calculated as (counts × 10^6^)/[length × sum of (counts/length)], and the m6A level was then measured by dividing the IP TPM by the input TPM. In addition, the normalized input TPM was regarded as the gene expression level. For the comparison among different stage, input count and IP count were normalized by TMM (trimmed mean of M-values) using R package “edgeR.” m6A methylation level was measured by dividing the IP TMM by the input TMM. The differential expressed (DE) genes between fetal and adult were identified by R package “edgeR” and only the *p*-valve < 0.05 with the absolute value of log2FC >1 were defined as DE genes. The criteria of differential methylated genes were the same with that of DE genes.

### m6A Peak Identification

The mapped reads of immunoprecipitation (IP) and Input libraries were provided for the macs2 (version 2.2.6) callpeak, with “--nomodel -g hs--keep-dup all--extsize 200” parameter to call m6A peaks. Only the peaks with more than two-fold enrichment compared to input and FDR <0.05 were kept. To further gain high confident peaks, we defined the peaks that had at least 50% overlap in two samples as true m6A peaks with the “−f 0.5” parameter of intersect function and then merged the peaks in different samples using bedtools (version 2.29.0).

### m6A Topological Distribution Analysis

To profile the topological distribution of m6A during development, we plotted the global distribution of m6As on mRNA using the R package “Guitar” (Cui et al., 2016). R package “ChIPseeker” (Yu, Wang, and He 2015) was used to annotate the m6A peaks with “annotatePeak” function. As some peaks may overlap with other regions, the priority of assignment was based on the following: “fiveUTRs,” “Promoters,” “threeUTRs,” “Exons,” and “Introns”.

To further explore the different topological distribution of m6A methylation between fetal and adult tissues, we clustered protein-coding genes into five groups based on the m6A distribution difference along genic region. The longest transcript of each gene was selected from R package “EnsDb.Hsapiens.v75.” Transcripts with 5′UTRs length <50 bp or 3′UTRs length <100 bp or CDS length <100 bp were filtered. In total 14191 genes were retained and used for downstream analysis. Then for each gene, the 5′UTRs, 3′UTRs, and CDS region was divided into 10, 20, and 20 windows, respectively. We normalized the peak density to the filtered genes’ windows using “normalizeToMatrix” function with “target_ratio = 1, k = 1” from R package “EnrichedHeatmap” (Gu et al., 2018). Last, we clustered the all genes into five groups with k-means method based on the normalized m6A peak density along transcripts of genes. The principle of k-means is calculating the distance of each object to the centroid of each class and then minimizing total within-cluster variances during iterative clustering process. According to this method, for genes with multiple peaks, the distances to each group will be calculated and then will be assigned to the group with the smallest distance.

### Tissue-specific Gene Expression Analysis

To analyze the tissue specificity difference among each group, we calculated tau using an online calculator (https://tspex.lge.ibi.unicamp.br/) to measure tissue specificity based on the TPM (Transcripts Per Kilobase of exon model per Million mapped reads) expression data. We downloaded the TPM expression data of representative 12 tissue types from Genotype-Tissue Expression (GTEx) database (https://www.gtexportal.org/home/), including breast, cerebellum, heart muscle, kidney, liver, lung, ovary, skeletal muscle, skin, spleen, stomach, and testis.

### Function Enrichment Analysis

Function enrichment analysis was performed by online tools metascape (https://metascape.org/gp/index.html), as well as R package “clusterProfiler” with MSigDB (Molecular Signatures Database), which collected several annotated gene sets including GO (Gene Ontology), KEGG (Kyoto Encyclopedia of Genes and Genomes), REACTOME, WIKIPATHWAYS, and hallmark. Adjusted *p* value <0.05 were used as significant cutoff. To find out whether 5′MGs were mainly responsible for the energy metabolism process, we download 381 energy metabolism associated genes from AmiGO (http://amigo.geneontology.org/amigo/search/ontology), including genes in terms of “respiratory electron transport chain,” “mitochondrial ATP synthesis coupled electron transport,” “electron transport chain,” “oxidative phosphorylation”.

### Potential Mechanisms Analysis of Different m6A Topology

To explored the binding preference of m6A Writer/Eraser/Reader (WER) among each gene group, the WER (*METTL3*, *YTHDF1*, *YTHDF2*, and *YTHDC1*) target genes were downloaded from the category of “Potential Targets (Binding)” from M6A2Target database (Deng et al., 2021). Due to the small numbers of the eraser target genes, only writer and reader target genes were used for our analysis.

Human genes’ structure information, including the length of 5′UTRs, the length of 3′UTRs, exon number, transcript length, transcript number were extracted from R packages “EnsDb.Hsapiens.v75”, and mouse genes’ structure information was extracted from R packages “EnsDb.Mmusculus.v79”.

To discover the sub-motifs of m6A peaks, findMotifsGenome.pl script of Homer（version 4.11.1）was used with “-len 6,8” parameters and all other parameters were set to the default settings. Based on the previous study, the RRACH motifs are widely divided into four sub-motifs (GGACH, AGACH, GAACH, and AAACH) (26). Therefore, we mainly focused on these four sub-motifs in peaks deposited in each genic regions for sub-motif analysis.

To obtain the conservation scores of each genic region, we first downloaded single base’s conservation score from UCSC table browser (http://hgdownload.cse.ucsc.edu/goldenPath/hg19/phastCons46way/vertebrate/). Then the exon and CDS region was retrieved from GENCODE GTF file, 3′UTRs and 5’UTR’s bed files were downloaded from The University of California Santa Cruz (UCSC) Table Browser (http://genome.ucsc.edu/cgi-bin/hgTables). Lastly, we mapped the single base signal to target regions by BEDOPS (version 2.4.39) with “bedmap” function and then the conservation score of target regions were estimated by the average value for all contained bases.

### Correlation Analysis Between Gene Expression and m6A Modification

To explore the relationship between m6A level and gene expression in each m6A topological gene group, Pearson correlation coefficients (*R*) between m6A level in specific genic region and gene expression was calculated within each group.

### m6A Topological Transition Analysis

The m6A topological transition patterns was visualized by R package “ggalluvial” and “ggplot2.” The time-specific genes during human development were downloaded from a previous study (Cardoso-Moreira et al., 2019), in which those genes with decreased expression during development were classified as “early” genes, and those with increased expression were classified as “late” genes. The human bivalent genes list was obtained from a previous study, which identified high-confidence bivalent genes based on several publicly available ChIP-seq datasets from hESC (Court and Arnaud 2017).

### Statistical Analysis

All statistical analysis were performed by R (v4.0.2). Tests involving comparisons among multi-groups were performed using Kruskal-Wallis rank-sum test or ANOVA, comparisons between two-groups were performed using Wilcoxon rank-sum test or *t*-test. Tests involving comparisons of number or ratio were performed using *z*-test, χ2 test or two-sided Fisher’s exact test. Results with a value of *p* value < 0.05 were considered statistically. ***p value < 0.001; **p value < 0.01; *p value < 0.05; ns, not significantly, *p* value > 0.05.

## Results

### Protein-Coding Genes Can be Classified Into Five Groups Based on m6A Distribution Along Genic Regions in Mammals

**FIGURE 1:**
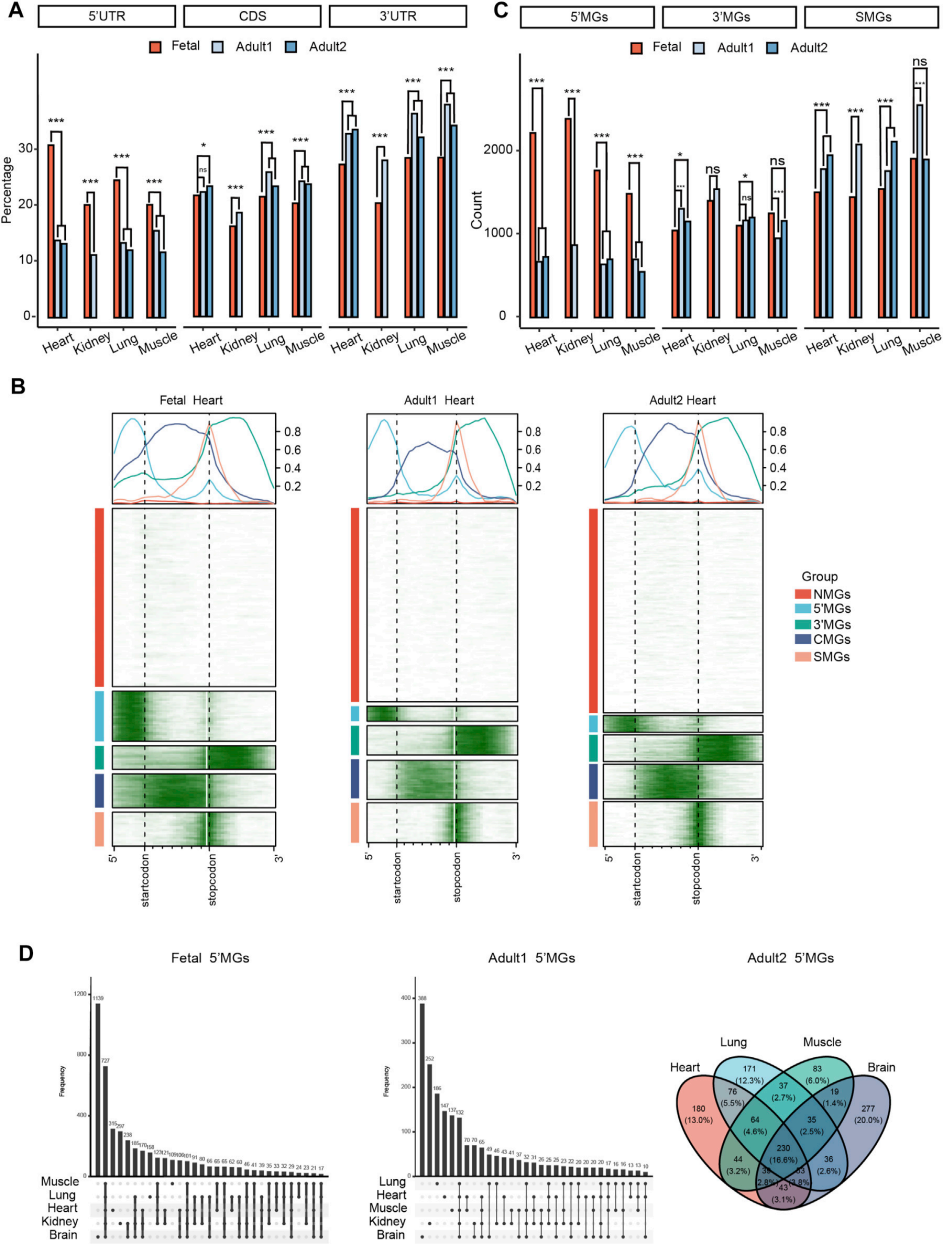
Different m6A topological patterns between human fetal and adult tissues. **(A)** The frequency of m6As located in the 5′ UTRs, CDS, and 3′ UTRs among human tissues in fetal and adult stages (*z*-test*,* ***p value < 0.001, *p value < 0.05, ns: not significantly). Adult1 means the first dataset of adult tissues and adult2 means the second datasets. **(B)** The m6A topological patterns along the m6A deposited regions in human fetal and adult heart tissues. We defined those five group genes as NMGs, 5′MGs, 3′MGs, CMGs, SMGs according to the m6A deposited regions. White color means without m6A peak, green color means with m6A peak. **(C)** The frequency of 5′MGs, 3′MGs and SMGs among human tissues in fetal and adult stages (*z*-test, ***p value < 0.001, *p value < 0.05, ns: not significantly). **(D)** The overlap of 5′MGs across all fetal (left) and adult (right) tissues.

To gain insight into the developmental regulation of m6A methylation during tissue development, we performed m6A methylome analysis in multiple human fetal and adult tissue samples. The raw m6A-seq data of representative human fetal and adult tissues were collected from previous studies (Xiao et al., 2019; Zhang, et al., 2020a; Liu et al., 2020), which provide the most comprehensive and high-quality m6A resource of human tissues to date. We used uniform pipeline to generate high-quality m6A peaks from raw data for all samples (see Methods for details). Tissues, namely, heart, kidney, lung, and muscle, were compared between fetal and adult stages. Tissues that were only available in one developmental stage were used for other analyses, including fetal placenta, fetal stomach, fetal liver, adult cerebellum, and adult spleen (see Methods for details). We first quantified the genic locations of the identified m6A peaks and found that the frequency of m6A peaks located in the 5′UTRs of fetal tissues was significantly higher than that of adult tissues (*p* value < 2.2e-16, *z*-test; Figure 1A). In contrast to the 5′UTRs, m6A around the 3′UTRs in fetal tissues had a lower density than that in adult tissues (*z*-test; Figure 1A). In other unpaired tissues, we found similar patterns with a higher m6A peak density and frequency of m6A peaks at the 5′UTRs in fetal tissues compared to adult tissues (Supplementary Figure S1A). These results demonstrated that fetal and adult tissues showed different m6A topological patterns in protein-coding mRNA transcripts. Given the m6A profiles of two adult datasets were generated using different antibodies and library construction methods (Supplementary Table S1), the observed consistent difference in both datasets suggested that the different m6A topological patterns between fetal and adult tissues is not due to batch effect caused by technical heterogeneity. Furthermore, we analyzed brain tissue which is composed of two independent datasets in both developmental stages, and found the similar pattern with a higher m6A peak frequency at the 5′UTRs and a lower frequency at the 3′UTRs in fetal tissues compared to adult tissues (Supplementary Figure S2A), again suggesting that the observed m6A topological difference between fetal and adult tissues was not affected by different experimental procedures.

Next, to systematically survey the m6A topological pattern in more detail, we clustered all the filtered protein-coding genes (see Methods for details) according to their m6A distribution along genic regions using the k-means algorithm (Supplementary Table S2). Strikingly, all the genes could be clearly classified into five groups in which m6A peaks were significantly enriched in different genic regions (Figure 1B; Supplementary Figures S1B–D, Supplementary Figure S2B). We defined the Group1 genes that showed no or little m6A modification as NMGs, Group 2 genes with m6A enriched mainly in 5′UTRs as 5′MGs, Group 3 genes with m6A enriched in 3′UTRs as 3′MGs, Group 4 genes with m6A enriched in the CDS as CMGs, and Group 5 genes with classical m6A enrichment around the stop codons as SMGs.

In general, the frequency of NMGs was the highest among these five groups across all tissues. Considering the m6A peaks located in 5′UTRs and 3′UTRs had the most significantly difference in two stages, we then compared the count of 5′MGs, 3′MGs, and SMGs among fetal and adult tissues. We observed the frequency of 5′MGs in fetal tissues was significantly higher than that in adult tissues (*p* value < 2.2e-16, *z*-test; Figure 1C, Supplementary Figure S2C, Supplementary Table S3) and SMGs had a significantly increase during tissue development. While 3′MGs had no consistently significant differences across tissues in fetal and adult. Next, we investigated whether the genes assigned to each group by k-means clustering were conserved among different tissues and found that the majority of genes in each group overlapped across most analyzed fetal and adult tissues (Figure 1D, Supplementary Figures S3A–D). To determine if the above observed different m6A topologies were conserved across species, we analyzed mouse tissues (adult brain, lung and heart, and fetal brain) and found that their protein-coding genes could also be clearly grouped into five groups based on the enrichment regions of m6A peaks, indicating that different m6A topological patterns among protein-coding genes are evolutionarily conserved in mammals (Supplementary Figure S1E, Supplementary Figure S2D, Supplementary Table S4).

Taken together, our results revealed that protein-coding genes can be classified into five groups based on m6A topological patterns. Fetal tissues had remarkably higher m6A density at 5′UTRs and lower density around stop codons than the corresponding adult tissues, which suggested that m6A in 5′UTRs of fetal tissues may contribute to embryonic development.

### Functional Characterization of Gene Groups With Different m6A Topologies

**FIGURE 2:**
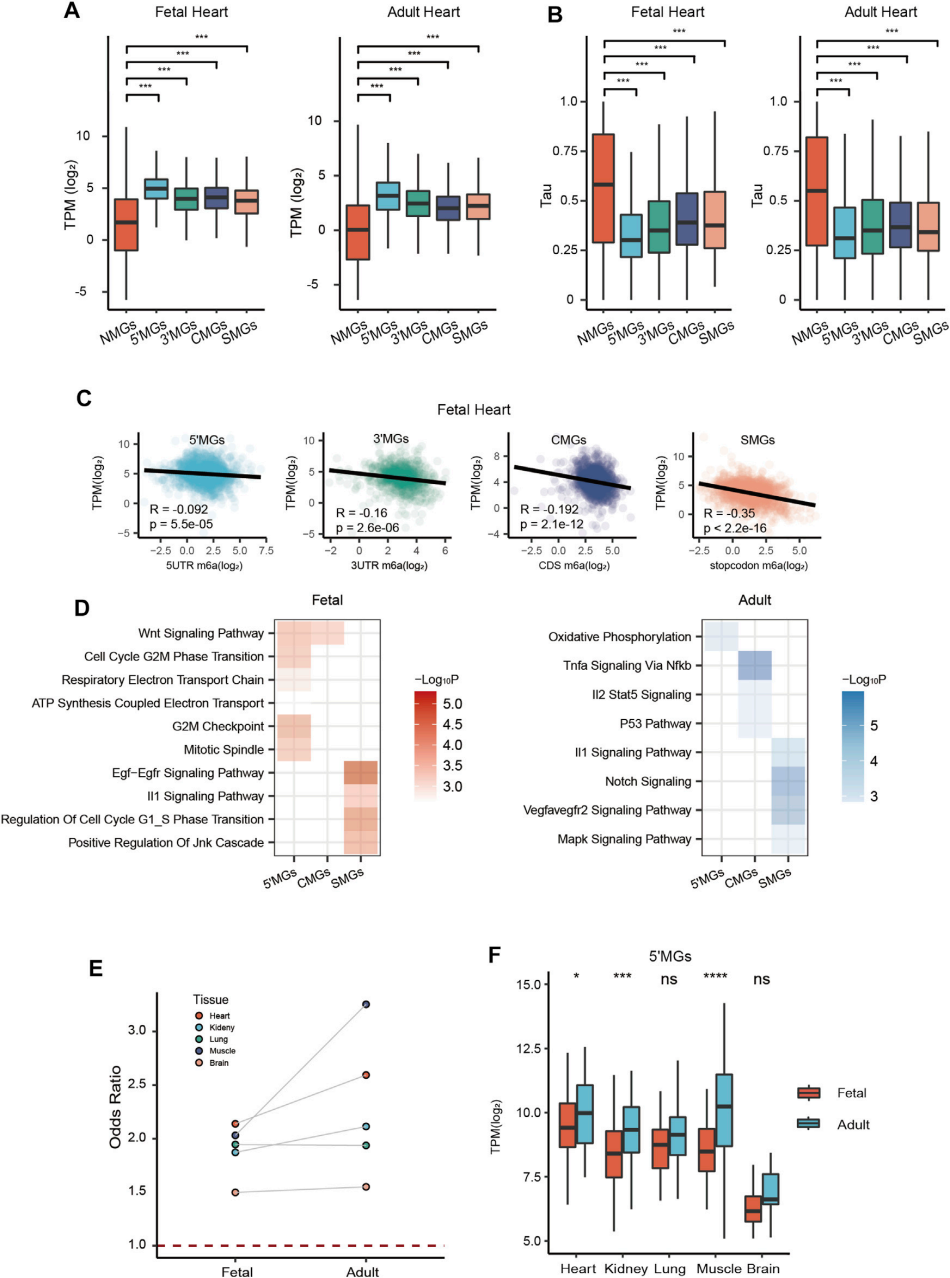
Functional characterization of gene groups with different m6A topologies. **(A)** The expression level (transcripts per kilobase per million mapped reads, TPM) of five group genes in fetal and adult heart tissue (ANOVA, ***p value < 0.001). **(B)** The tissue specificity score tau of five gene groups in fetal and adult heart tissue (Kruskal–Wallis rank-sum test, ***p value < 0.001). **(C)** Scatter plots showed the correlation analysis between expression level and m6A methylation at different genic regions, including 5′UTRs, 3′UTRs, CDS, and around stop codon (100 nucleotides upstream and 100 nucleotides downstream of the stop codon) in fetal heart tissue. **(D)** Heatmap showing function enrichment analysis of each group genes across all tissues in fetal (Left) and adult (Right) stages. Color key from white to red indicates −Log*10P* in fetal tissues and from white to blue indicates −Log*10P* in adult tissues. **(E)** The odds ratio of 5′MGs which belonged to the energy metabolism associated GO term genes across all tissues in fetal and adult stages. **(F)** The expression level of energy metabolism associated 5′MGs across all human tissues in fetal and adult stages (Student’s *t*-test, ***p value < 0.001, *p value < 0.05, ns: not significantly).

To explore the biological functions of the above five identified gene groups, we first examined gene expression levels among different groups and found that NMGs had the lowest expression level compared to the other groups across all five paired tissues in fetal and adult stages (*p* value < 2e-16, one-way analysis of variance (ANOVA); Figure 2A, Supplementary Figure S4A). Then, to examine the tissue specificity of gene expression among different groups, we calculated the tau value for genes in each group. Tau is widely used to measure tissue specificity (Kryuchkova-Mostacci and Robinson-Rechavi 2017). It varies from 0 to 1, where 0 means broadly expressed, and 1 means tissue specific. We found that NMGs had a significantly higher tau value than genes with m6A modification in other groups (*p* value < 2.2e-16, Kruskal–Wallis rank-sum test; Figure 2B, Supplementary Figure S4B), suggesting that NMGs are prone to be tissue-specific genes, which is consistent with above observed low expression level of NMGs and also consistent with previous studies which showed that tissue-specific genes usually have lower expression (Kryuchkova-Mostacci and Robinson-Rechavi, 2015; Kryuchkova-Mostacci and Robinson-Rechavi, 2017). The relatively low tau values of genes with m6A modification also suggested that ubiquitous genes are more likely be m6A regulated, consistent with the previous studies (He et al., 2019). We further explored the relationship between the m6A level and gene expression for each group. Interestingly, we observed that the m6A methylation level in each group showed a significant negative correlation with gene expression across all tissues (Figure 2C, Supplementary Figure S5). This result indicated that there may be different mechanisms for regulating gene expression for gene groups with and without m6A modification.

Next, to characterize functions for each group, we performed functional enrichment analysis using the Molecular Signatures Database (MSigDB), which includes several annotated gene sets, including widely known Gene Ontology (GO) and Kyoto Encyclopedia of Genes and Genomes (KEGG) pathways. Since our above results showed large gene overlaps across different tissues for each group, we identified overlapping genes across tissues within each group in fetal and adult stages and then used them for our downstream analysis.

In general, we observed that in both developmental stages, 5′MGs were strongly associated with functional terms related to energy metabolism, such as “ATP synthesis coupled electron transport” and “oxidative phosphorylation.” CMGs and SMGs were significantly enriched in signaling pathways related to growth and development, such as “WNT signaling,” “EGF-EGFR signaling pathway,” and “NOTCH signaling” (Figure 2D). However, for 3′MGs, we found few significantly enriched functional terms.

Given that 5′MGs were strongly associated with energy metabolism in both stages, we calculated the odds ratio of the energy metabolism-associated genes in 5′MGs compared to these genes in all other groups. We found that the odds ratios were all greater than 1 across all tissues, and adult muscle had the highest odds ratio (Figure 2E, *p* value < 0.05, χ2 test). More importantly, the gene expression of fetal 5′MGs related to energy metabolism was significantly or marginally upregulated in the adult stage compared to the fetal stage across almost all human tissues (Figure 2F, *p* value < 0.05, *t* test). This is consistent with a previous study showing that the expression of mitochondrial proteins could be increased (Darby et al., 2020) and the oxidative phosphorylation is the main way to produce ATP during postnatal development (Piquereau and Ventura-Clapier 2018).

Our above results revealed that genes with the same m6A topology largely shared similar functions between fetal and adult tissues among human tissues, but we also observed some developmental stage-specific differences in some groups. For example, fetal 5′MGs and fetal SMGs were significantly enriched in cell cycle processes, such as “cell cycle g2_m phase transition” and “regulation of cell cycle G1_S phase transition”, but adult 5′MGs and adult SMGs were not enriched in these processes (Figure 2D), which suggested that genes with m6A methylation at 5′UTRs and around stop codons could regulate cell cycle processes within specific developmental stages.

In summary, our results demonstrated that genes in five groups with different m6A topologies had distinct biological functions. The functions of 5′MGs were linked with energy metabolism. CMGs and SMGs had a similar enrichment in signaling pathway-associated terms related to cell growth and differentiation, respectively.

### Potential Mechanisms Associated With Different m6A Topologies Among Different Gene Groups

To investigate the potential mechanisms of different m6A topologies among genes, we performed a systematic survey regarding the gene structure and biological functions of different groups.

**FIGURE 3:**
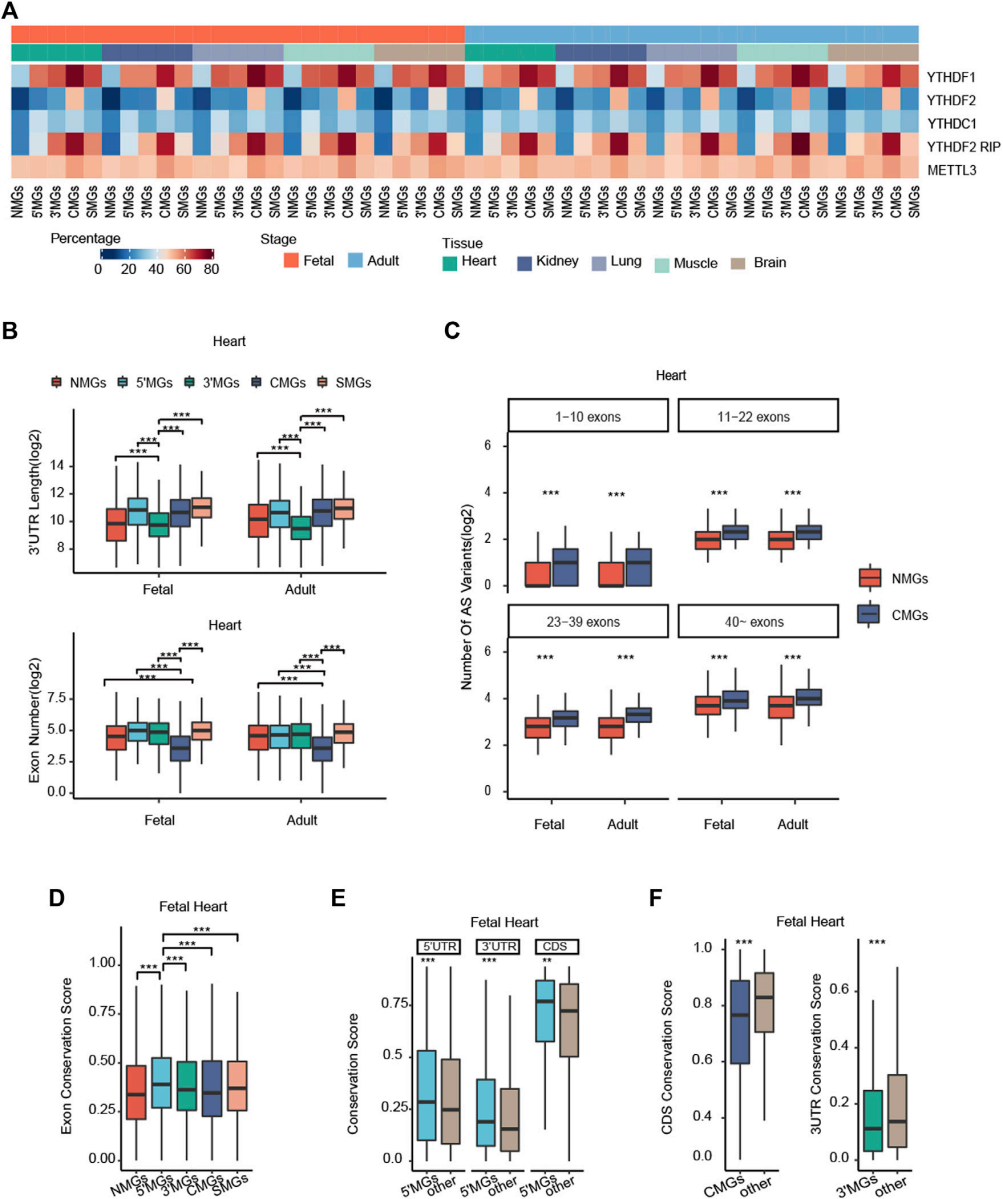
Potential mechanisms associated with different m6A topologies among different gene groups. **(A)** Heatmaps showing the distribution of *YTHDF1*, *YTHDF2*, *YTHDC1*, *YTHDF2* (RIP), and *METTL3* targeted genes in each group across all human tissues in fetal and adult stages. Colors represent the percentage of WER targeted genes. **(B)** The 3′UTRs length and exon number of each gene group among human heart tissue in fetal and adult stages (Kruskal–Wallis rank-sum test, ***p value < 0.001). **(C)** The number of alternative splicing (AS) variants in NMGs and CMGs with distinct exon numbers in human fetal and adult heart tissue (Wilcoxon rank-sum test, ***p value < 0.001). **(D)** The exon conservation score of five gene groups in fetal heart tissue (Kruskal–Wallis rank-sum test, ***p value < 0.001). **(E)** The conservation score of 5′UTRs, 3′UTRs, and CDS among 5′MGs and other group genes with m6A methylation of human fetal heart tissue (Wilcoxon rank-sum test, ***p value < 0.001, **p value < 0.01). **(F)** The CDS conservation score of CMGs and other group genes with m6A methylation (Left); the 3′UTRs conservation score of 3′MGs and other group genes with m6A methylation in human fetal heart tissue (Right) (Wilcoxon rank-sum test, ***p value < 0.001).

First, to explore whether the binding of m6A writers/readers contributes to the m6A topology, we obtained target genes of m6A writers and readers from M6A2Target, which is the most comprehensive database for the target genes of writers, erasers and readers (WER) of m6A modification validated by low-throughput experiments or indicated by high-throughput sequencing (Deng et al., 2021). We compared the distribution of these target genes among the five gene groups. As expected, NMGs showed the lowest proportion of target genes of m6A readers. Furthermore, our results revealed that the target genes of the m6A readers *YTHDF1* and *YTHDF2* were preferentially enriched in CMGs, and *YTHDC1*-targeted genes were enriched mainly in 5′MGs and CMGs compared to other groups of genes among all tissues (*p* value < 2.2e-16, χ^2^ test; Figure 3A). To validate and confirm the above results, we further conducted the same analysis using RIP-seq data of *YTHDF2* from a published study (Fang et al., 2021) and showed a similar result (Figure 3A). Then, we also examined the distribution of m6A writer *METTL3*-targeted genes in each group and found that these target genes were significantly enriched in CMGs compared to other group genes in all analyzed tissues except for adult lung (*p* value < 0.05, χ2 test; Figure 3A), which is consistent with a previous study showing that most *METTL3*-dependent m6A occurs within the coding region of *METTL3*-bound gene transcripts (Barbieri et al., 2017). Taken together, our results suggested that the different binding preferences of m6A readers and writers may have a significant effect on m6A deposition among different genic regions.

Then, to determine whether the structural properties of genes could affect m6A topology, we analyzed the length of the 5′UTRs, the length of the 3′UTRs, and the exon number of the genes in each group. We found that the average 3′UTRs length of 3′MGs was significantly shorter than that of the other groups and that the exon number of CMGs was significantly less than that of the other groups among all mammalian tissues (*p* value < 0.05, Kruskal-Wallis rank-sum test, Figure 3B, Supplementary Figure S6A). However, the average 5′UTRs length of 5′MGs and CDS length of CMGs were not significantly higher or lower than that of the other gene groups (Supplementary Figure S6A, Supplementary Figure S7A). Given that the 3′UTRs contains several regulatory elements for posttranscriptional regulation (Xin, Hu, and Kong 2008; Xu et al., 2019), these results suggested that m6A may add an additional layer of gene regulation and could compensate for the regulation of gene expression for those genes with shorter 3′UTRs. Similarly, because alternative splicing (AS) is an important mechanism for increasing regulatory complexity and is positively correlated with the exon number of genes (Morata et al., 2013), we hypothesized that m6A regulation could increase AS levels in CMGs with low exon numbers. To test this hypothesis, we compared the AS variant numbers (measured by total number of annotated transcripts per gene) between the NMGs and CMGs in stratified groups with similar exon numbers. Supporting our hypothesis, we found that the AS variant number in CMGs was significantly greater than that in NMGs in each stratified group across all tissues (*p* value < 0.01, Wilcoxon rank-sum test, Figure 3C, Supplementary Figure S6B). In addition, to confirm that the gene structure characteristics of each m6A topology group are conserved in mammals, we conducted similar analyses in mouse tissues. We also found that the length of the 3′UTRs of 3′MGs and the exon number of CMGs were significantly lower than those of other group genes (Supplementary Figure S7B). Overall, our results revealed that the gene structure characteristics of 3′MGs and CMGs are conserved in mammals and that m6A methylation may act as an additional regulatory layer for increasing regulatory complexity.

Given that m6A peaks are enriched in the consensus motif RRACH, we next asked whether the composition of submotifs was different among genic regions. Our results showed that all the m6A peaks in the 3′UTRs, CDSs, and 5′UTRs were more likely to be found in the GGACH submotif (Supplementary Figure S6C), which is consistent with a previous study showing that the most significantly enriched motifs in m6A peaks were characterized by the canonical GGACH (H=U/A) sequence (Li et al., 2019; Xue et al., 2021).

Finally, we investigated the relationship between evolutionary conservation and different m6A topologies. To evaluate the conservation of each group of genes, we employed PhastCons to calculate the gene conservation score (see Methods for details), and the more conserved gene showed the higher conservation score. Interestingly, we found that among the five groups, 5′MGs showed the highest conservation score, whereas NMGs showed the lowest score across all tissues (*p* value < 2.2e-16, Kruskal–Wallis rank-sum test; Figure 3D, Supplementary Figure S8A), which is consistent with a previous study showing that sites with m6A methylation showed higher evolutionary conservation than other regions (Meyer et al., 2012). In line with this, 5′MGs with the highest conservation score also showed the lowest tau value among all the groups. To further clarify whether the different conservation among the four m6A-modified gene groups was attributed to their m6A topologies, we compared the conservation score for each genic region among the groups. Given that the conservation score of 5′MGs was the highest compared to those of the other groups, we first explored the association between conservation and m6A deposition in 5′UTRs. Indeed, we observed that the conservation score of 5′UTRs in fetal but not adult 5′MGs was significantly higher than that in other group genes across all tissues (*p* value < 2.2e-16, Wilcoxon rank-sum test, Figure 3E, Supplementary Figure S8B), which indicated that these conserved genes may play important roles, especially in the fetal stage. This again suggested that fetal 5′MGs could be linked to early development. However, the conservation scores of the 3′UTRs and CDS in fetal and adult 5′MGs were also significantly higher than those in other groups of genes across all tissues (*p* value < 2.2e-16, Wilcoxon rank-sum test, Figure 3E, Supplementary Figure S8B), which suggested that the high conservation level of fetal 5′MGs was not associated with its m6A topology. Moreover, we also found that the conservation score of the 3′UTRs in 3′MGs and the conservation score of the CDS in CMGs were significantly lower than those of other group genes (*p* value < 2.2e-16, Wilcoxon rank-sum test, Figure 3F, Supplementary Figure S8C), which further confirmed that the distinct conservation among the groups was not directly associated with m6A topology.

Overall, our results revealed that genes in the five groups with different m6A topologies had distinct characteristics, including shorter 3′UTRs for 3′MGs, fewer exon numbers for CMGs, and higher evolutionary conservation for 5′MGs.

### Dynamic m6A Topological Transition During Mammalian Tissue Development

**FIGURE 4:**
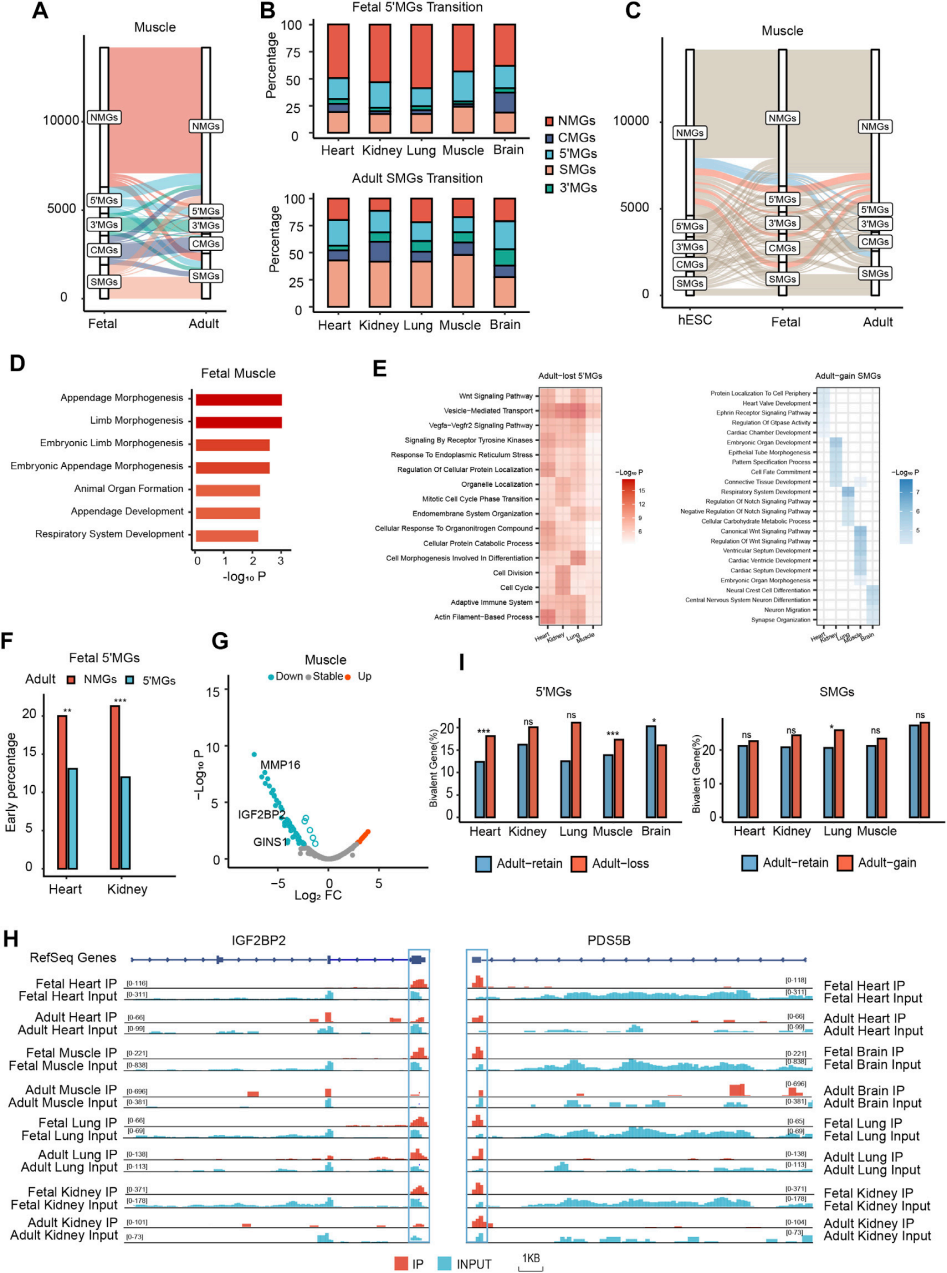
Dynamic m6A topological transition during mammalian tissue development which was associated with tissue development. **(A)** The m6A topological transition between human muscle tissue in fetal and adult stages. **(B)** The percentage of fetal 5′MGs transited to adult gene groups and the percentage of adult SMGs transited from fetal gene groups across all human tissues in fetal and adult stages. **(C)** The transition between three developmental stages, hESC, fetal, and adult of human muscle tissue. **(D)** Bar plot showing function enrichment analysis of muscle fetal-specific m6A genes. Color key from white to red indicates −Log*10P*. **(E)** Heatmap showing function enrichment analysis of adult-loss 5′MGs (Left) and adult-gain SMGs (Right) across tissues. Color key from white to red indicates −Log*10P* in adult-loss 5′MGs and from white to blue indicates −Log*10P* in adult-gain SMGs. **(F)** The distribution of early genes in adult-loss and adult-retain 5′MGs across human heart and kidney tissues (χ2 test, ***p value < 0.001, **p value < 0.01). Those genes with significant decreased expression during development were classified as “early” genes. **(G)** Volcano plot showing the distribution of all differentially expressed adult-loss 5′MGs during muscle development. LogFC means the fold change (log2) of expression level during tissue development. **(H)** Integrated genome (IGV) browser views of representative genes (*IGF2BP2* and *PDS5B*) with 5′UTRs m6A loss and expression down during tissue development across human tissues in fetal and adult stages. **(I)** The distribution of bivalent genes in the adult-loss 5′MGs (Left) and adult-gain SMGs (Right) (Two-sided Fisher’s exact test, ***p value < 0.001, *p value < 0.05, ns: not significantly).

Since our results showed that there were significantly different m6A topological patterns between human fetal and adult tissues, especially for 5′MGs and SMGs, we next systematically investigated the m6A topological transition during tissue development. To measure the transition among different m6A topological groups quantitatively, we first defined the overlap ratio for each group as the proportion of genes overlapping between the fetal and adult tissues among genes of each group in fetal or adult stages. In general, NMGs were the most conserved group regarding m6A topology with the highest overlap ratio (65.28%–73.65%), while 5′MGs were the most dynamic group with the lowest overlap ratio (13.99%–23.35%), and SMGs also had a low overlap ratio (28.69%–37.79%) (Figure 4A, Supplementary Figure S9A). Next, to examine the transition pattern of each group during development in detail, we defined transition ratio for each group as the proportion of genes transited between different groups from fetal to adult using fetal tissues as reference (Supplementary Table S5). For 5′MGs, approximately 50% of fetal 5′MGs transitioned to adult NMGs (defined as adult-loss 5′MGs), which is remarkably higher than other transition types (*p* value < 2.2e-16, *z*-test; Figure 4B), implying a significant loss of m6A methylation in 5′UTRs during tissue development. In addition, we found a significant increase in adult SMGs compared to matched fetal tissues, and approximately half of the adult SMGs transitioned from other fetal m6A topological groups (defined as adult-gain SMGs) (Figure 4B, Supplementary Table S6). And in mouse brain tissues, we observed a similar transition pattern (Supplementary Figure S9B). These results revealed that 5′MGs and SMGs were the most dynamic gene groups in m6A topology during tissue development. In summary, our results revealed extensive m6A topological changes between fetal and adult tissues, indicating high plasticity of the m6A topology of mRNAs and potentially important roles of topological transitions during tissue development.

### m6A Topology in hESCs and its Transition During Human Embryogenesis

To further determine whether these m6A topological transitions occurred at an earlier stage of development, we collected raw m6A-seq data from two human embryonic stem cell (hESC) samples from a previous study and processed the data with the same pipeline (Batista et al., 2014). First, all protein-coding genes in hESCs were also clustered into five groups, as shown in our above results. We found that the number of 5′MGs in hESCs was less than that in fetal tissues but higher than that in adult tissues (Supplementary Figure S9C, Supplementary Table S3). Then, we found that a certain number of 5′MGs in hESCs transitioned to fetal NMGs (Supplementary Table S7, Supplementary Table S8), which is consistent with the transition trends between fetal and adult tissues. Through comparisons of m6A methylomes among three stages, we identified fetal- and adult-specific m6A-modified genes. Remarkably, we found that fetal-specific m6A-modified genes were predominantly enriched in fetal 5′MGs in all analyzed tissues (Figure 4C, Supplementary Figure S9D, Supplementary Table S9). In contrast, there was no significant distribution preference for adult-specific m6A-modified genes (Supplementary Table S9). This result suggested that the m6A topology of fetal 5′MGs was developmental stage specific, transiently shaped during embryogenesis and then lost in adult tissues.

Furthermore, we performed functional enrichment on these stage-specific m6A-modified genes and found that these genes were associated with stage-specific functions in certain tissues. For example, in muscle, fetal-specific m6A-modified genes were significantly associated with embryonic development (Figure 4D), such as “embryonic limb morphogenesis” and “animal organ formation”. Additionally, adult-specific genes in muscle 5′MGs were remarkably related to energy metabolism and tissue-associated progress (Supplementary Figure S9E), such as “oxidative phosphorylation” and “skeletal muscle contraction”. Altogether, these findings suggested that m6A topology transition existed at an earlier stage and that the biological function of stage-specific 5′MGs was closely linked to tissue development.

### Genes With m6A Topological Transitions Between Fetuses and Adults Were Associated With Tissue Development

To further address the functional association between fetal-to-adult m6A topological transitions and tissue development, we focused our analysis on fetal 5′MGs and adult SMGs, which showed the most dramatic topological changes.

First, to examine the biological function of 5′MGs and SMGs with m6A topology transition during human tissue development, we performed functional enrichment analysis for adult-loss 5′MGs and adult-gain SMGs. In most tissues, adult-loss 5′MGs were also functionally enriched in GO terms related to cell cycle processes, such as “mitotic cell cycle phase transition,” “cell division,” and “cell cycle” (Figure 4E), which suggested that adult-loss 5′MGs may affect tissue development by regulating cell cycle processes. In brain tissues, we found these genes linked to “Synaptic Vesicle Cycle” functions (Supplementary Figure S9F). Furthermore, adult-gain SMGs were dramatically enriched in tissue development-associated GO terms, such as “heart valve development,” “connective tissue development,” “respiratory system development” and “central nervous system neuron differentiation” (Figure 4E).

Then, we analyzed the relationship between m6A topological transitions and the developmental stage specificity of gene expression. We obtained “early” and “late” developmental stage-specific genes from a previous study that comparatively analyzed the transcriptomes of multiple types of tissues across several developmental time points from the early embryonic stage to adulthood among seven species, including humans (Cardoso-Moreira et al., 2019). We used shared tissues between two datasets (heart and kidney) for further analysis and redefined the early and late developmental stage-specific genes as fetal- and adult-specific genes, respectively. Intriguingly, we found that adult-loss 5′MGs were significantly enriched in fetal-specific genes compared to adult-retained 5′MGs (*p* value < 0.01, *χ* ^2^ test; Figure 4F), which indicated that fetal-specific developmental genes tend to exhibit m6A in 5′UTRs in fetuses and lose their m6A modifications during the tissue differentiation process. Then, we analyzed the gene expression in adult-loss 5′MGs and found that the expression of these genes was significantly downregulated in adult compared to fetal tissues (*p* value < 0.05, *z*-test; Figure 4G, Supplementary Figure S10A). For example, among these downregulated adult-loss 5′MGs in most tissues, insulin-like growth factor 2 mRNA binding protein 2 (*IGF2BP2*) is a critical maternal activator in early zygotic genome activation, and maternal deletion of *IGF2BP2* can cause embryonic arrest at the 2-cell stage (Liu et al., 2019). In addition, the cell population proliferation gene PDS5 cohesin-associated factor B (*PDS5B*) is also essential for mammalian development (Figure 4H). *PDS5B*-deficient mice were reported to die shortly after birth and exhibit multiple congenital anomalies, including heart defects, cleft palate, and germ cell depletion (Zhang et al., 2007). However, adult-gain SMGs did not show significant enrichment in adult-specific developmental genes compared to adult-retained SMGs in all analyzed tissues (*p* value = 0.52, 0.61, *χ* ^2^ test; Supplementary Figure S10B). In summary, our results suggested that the expression of adult-loss 5′MGs was significantly downregulated via m6A topological transitions and was critical for tissue development.

Previous studies have revealed that bivalent genes marked with both active (H3K4me3) and repressive (H3K27me3) histone modifications play an important role in cell differentiation and tissue development (Vastenhouw and Schier 2012). To further understand the function of m6A topological transitions on tissue development, we asked whether genes with m6A topological transitions were also associated with bivalent genes. We acquired the bivalent genes from a previous study, which summarized a high-confidence bivalent gene list based on several publicly available ChIP-seq datasets from hESCs (Court and Arnaud 2017). Then, we found that bivalent genes were enriched in the adult-loss 5′MGs and that the proportion was significantly higher than that of adult-retained 5′MGs in most tissues (*p* value < 0.05, two-sided Fisher’s exact test; Figure 4I). We also found that there was a marginally significantly higher proportion of adult-gain SMGs than adult-retained SMGs in most tissues (Figure 4I).

Taken together, our results revealed that the genes with m6A loss in the 5′UTRs during tissue development were strongly associated with developmental stage-specific genes and that these genes had an intimate connection with early tissue development by regulating the cell cycle process. Additionally, the genes with m6A gain in stop codons during development are significantly associated with tissue-specific functions.

### m6A Transition Between Normal and Cancer Tissues Related to Embryonic Development

**FIGURE 5:**
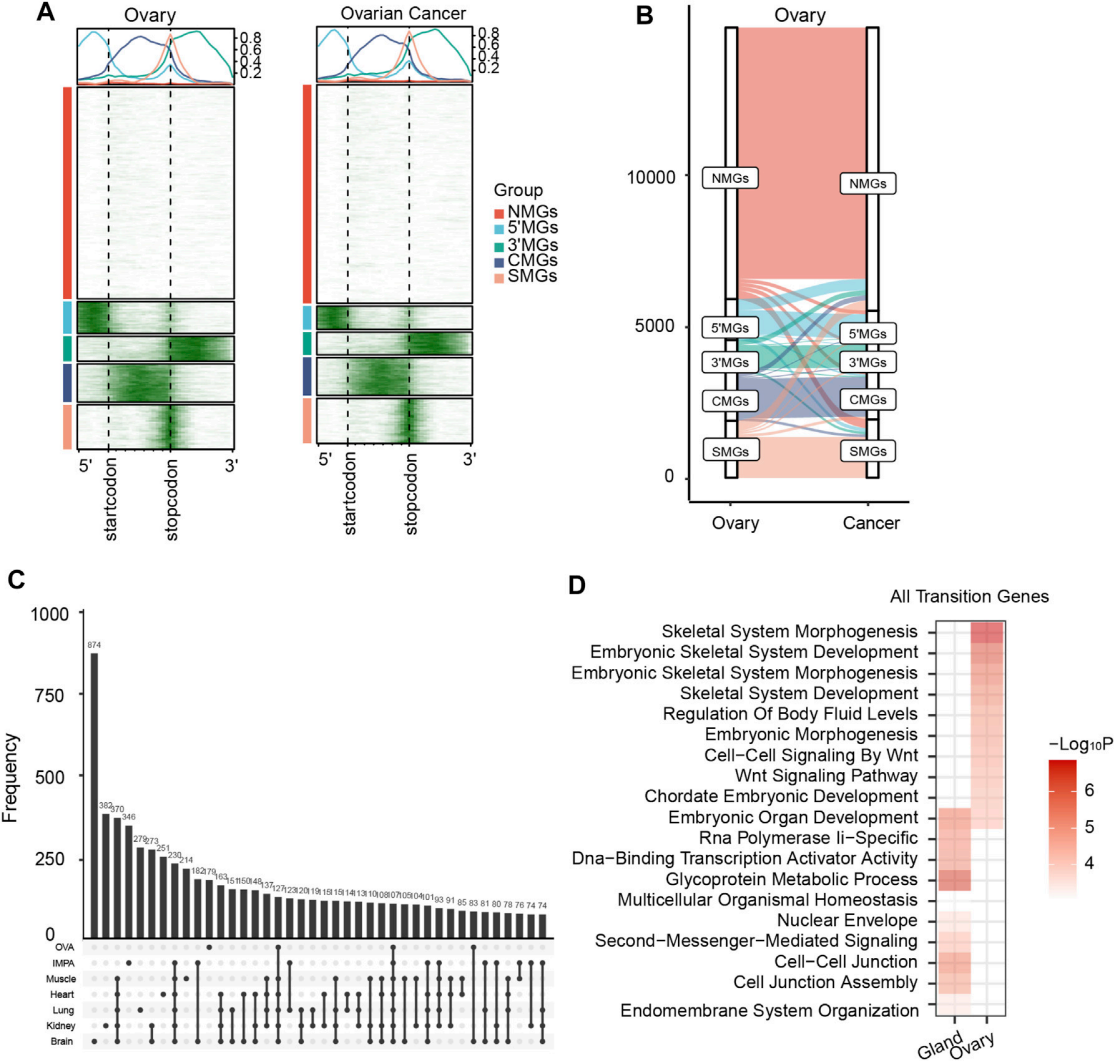
m6A transition between normal and cancer which related to embryonic development. **(A)** The m6A topological patterns along the m6A deposited regions in human ovary and ovarian cancer. White color means without m6A peak, green color means with m6A peak. **(B)** The m6A topological transition between the ovary and ovarian cancer. **(C)** The overlap of all transited genes among all human tissues, invasive malignant pleomorphic adenoma (IMPA) and ovarian (OVA) cancer samples. **(D)** Heatmap showing function enrichment analysis of all transited genes between the two normal and cancer samples. Color key from white to red indicates −Log*10P*.

It is well acknowledged that abnormal expression of developmental genes may promote cancer progression (Stewart et al., 2016). Therefore, we asked whether there was an m6A topological transition during carcinogenesis. We collected and analyzed m6A-seq data from two types of cancer, namely, invasive malignant pleomorphic adenoma (IMPA) and ovarian cancer (Zhang et al., 2019; Han et al., 2021). As expected, all protein-coding genes were clustered into five different groups for both cancer and matched normal tissues (Figure 5A, Supplementary Figure S10C). Remarkably, we found a similar m6A topological transition pattern during cancer progression to that during tissue development. For example, compared to normal tissues, tumor samples also showed a significant loss of 5′MGs and gain of new SMGs (Figure 5B, Supplementary Figure S10D). More importantly, we obtained cancer-transiting genes which with m6A topological transitions from normal to cancer tissues. And we found a large overlap of these cancer-transiting genes with all the above identified genes with transitions during tissue development, which not only confirmed the close relationship between tissue development and cancer progression but also emphasized the critical role of the m6A topological transition in cell differentiation (Figure 5C). Furthermore, we performed GO enrichment analysis to analyze the biological functions of genes transitioning between normal and cancer tissues. The results showed that genes with a transition from normal to tumor were strongly enriched in embryonic development-associated processes in both cancer types, such as “embryonic organ development” and “embryonic morphogenesis” (Figure 5D). These results suggested that the m6A topological transition might be a universal mechanism involved in the regulation of many biological processes and could play important roles in cellular differentiation and lineage commitment.

## Discussion

The current understanding of m6A modification in mammalian development is mainly focused on the landscapes in different stages and the regulation of gene expression. However, the dynamic change in the m6A topological pattern during mammalian development and the underlying mechanism are largely unknown. In this study, we revealed five gene groups with distinct m6A topologies and biological characteristics based on m6A deposition along the genic region. Strikingly, we observed extensive m6A topological transitions during both tissue development and tumorigenesis. More importantly, these genes with topological transitions were strongly associated with embryonic development and tissue-specific functions. Collectively, our results uncovered the critical role of dynamic changes in m6A topology in the developmental regulation of gene expression.

Our results revealed the functions and biological characteristics of these five groups with distinct m6A topologies. First, we observed that 3′MGs had shorter lengths of 3′UTRs and that their m6A methylation level was negatively correlated with gene expression. Consistent with these results, the m6A methyltransferase component VIRMA mainly contributed to mediating m6A deposition in 3′UTRs and could result in the shortened length of 3′UTRs via the selection of proximal APA (Yue et al., 2018), indicating that m6A modifications in 3′UTRs were significantly associated with the length of 3′UTRs of transcripts. Furthermore, given that the 3′UTRs of genes contain multiple regulatory elements, including miRNA-binding sites, the m6A methylation level of the 3′UTRs site could affect mRNA stability or regulate gene expression through human antigen R and microRNA pathways, resulting in a decrease in the mRNA expression level (Wang et al., 2014b). This implied that m6A methylation enriched in the 3′UTRs could regulate gene expression by controlling the length of the 3′UTRs of transcripts. Then, we found that CMGs had fewer exon numbers and more WER binding preference as well as more AS types than NMGs with the same exon number. Thus, we proposed a hypothesis that m6A modification in the coding region could regulate the AS process by recruiting different WERs. Supporting this hypothesis, previous studies showed that *ALKBH5* could regulate the AS process by decreasing the m6A level of the splicing sites in transcripts, thereby affecting the expression of different gene transcripts (Tang et al., 2018). Moreover, the m6A reader *YTHDC1* was reported to regulate mRNA exon splicing by binding to the splicing factor *SRSF3* (Kasowitz et al., 2018). Finally, we found that *YTHDC1* preferred to target m6A modifications in the 5′UTRs and that the m6A level of 5′MGs was negatively related to gene expression, indicating that m6A modification in the 5′UTRs may affect gene transcription *via* the binding of *YTHDC1*. Taken together, we suggest that m6A modifications at different genic regions regulate mRNA transcription in a multilayered way, exerting diverse biological functions.

We reported extensive m6A topological transitions between fetal and adult stages among different tissues, especially for the loss of 5′MG and the gain of SMG. Consistent with this finding, the distribution of m6A modifications along the genic region in mice was significantly related to cerebellar development (Ma et al., 2018). More importantly, gene expression analysis revealed significant downregulation of adult-loss 5′MGs, including many genes associated with early tissue development. For example, Go-Ichi-Ni-San (GINS) complex subunit 1 (*GINS1*, also known as *PSF1*) is essential for eukaryotic DNA replication, and homozygous null mutations of GINS component-encoding genes are embryonic lethal in mice (Cottineau et al., 2017). In addition, recent studies also confirmed that the LDL receptor-related protein 6 (*LRP6*) gene was crucial for the development of several tissues, including the mammary gland (Zhong et al., 2012), skeleton (Joeng et al., 2011) and intestine (Lindvall et al., 2009). Based on previous results that m6A modification in 5′UTRs could promote translation mediated by eIF3 (Coots et al., 2017), we suggested that m6A topological changes in 5′UTRs, especially in adult-loss 5′MGs, could play an important role in regulating these key developmental genes by reducing their expression. In addition, adult-gain SMGs in different tissues were significantly related to tissue-specific functions. For example, among the adult-gain SMGs in the heart, we found that T-Box Transcription Factor 5 (*TBX5*) was highly expressed in heart tissue, and the overexpression of *TBX5* inhibited myocardial growth and trabeculation *in vitro* and *in vivo*, which resulted in congenital cardiac septation defects (Hatcher et al., 2001; Garg et al., 2003). In addition, for the adult-gain SMGs in the lung, we noticed that *PDGFRA* was necessary to build the gas exchange surface area, ensuring the function of the respiratory system for lung tissue development (Hatcher et al., 2001). Overall, our results demonstrated that dynamic m6A topological transition could be a signature during mammalian tissue development and could alter the expression of development-related genes through a variety of molecular mechanisms.

In summary, we identified and characterized five gene groups with distinct m6A topological patterns. Moreover, we revealed dramatic m6A topological transitions during tissue development, providing new insights into the regulatory role of m6A in mammalian development.

## Data Availability

The datasets presented in this study can be found in online repositories. The names of the repository/repositories and accession number(s) can be found in the article/Supplementary Material.
