# Supplementary material for: m6A Topological Transition Coupled to Developmental Regulation of Gene Expression During Mammalian Tissue Development
Source: Front Cell Dev Biol. 2022 Jul 5;10:916423. doi: 10.3389/fcell.2022.916423 (PMC9294180; doi:10.3389/fcell.2022.916423)
Supplement: Supplementary file 1 [file DataSheet1.zip › Additional Files/Supplementary Figure.docx]

**
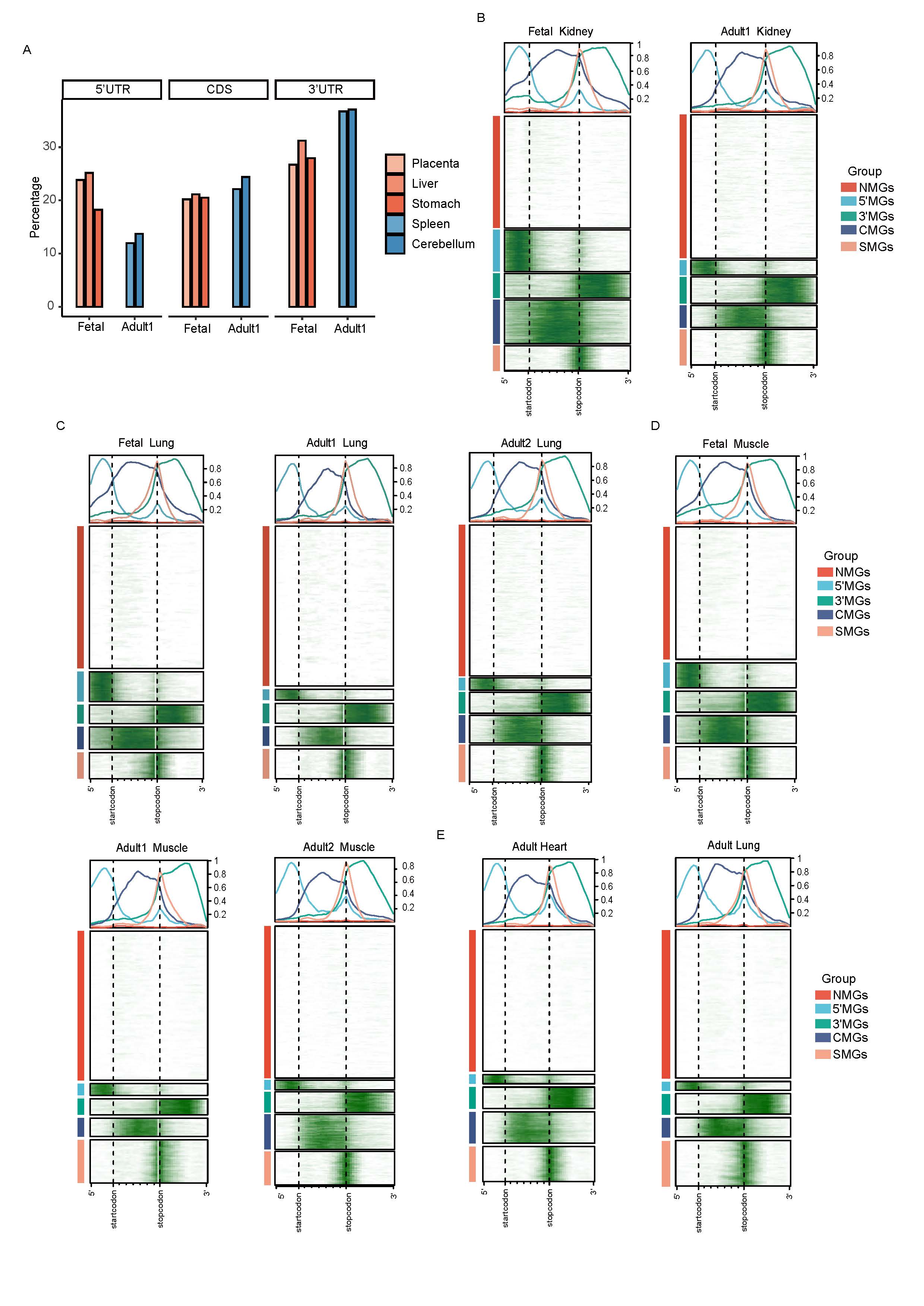
**

**Supplementary Figure 1. m6A profile in human and mouse tissues.**  **(A)** The frequency of m6As located in the 5′ UTRs, CDS and 3′ UTRs among unpaired five human tissues. **(B-D)** The m6A topological patterns along the m6A deposited regions for human kidney, lung, muscle tissues in fetal and adult stages. Adult1 means the first dataset of adult tissues and adult2 means the second datasets. NMGs were defined as genes with no or little m6A modification, 5′MGs were defined as genes with m6A mainly enriched in 5′UTRs, 3′MGs were defined as genes with m6A preferred to enrich in 3′UTRs, CMGs were defined as genes in which m6A enriched in CDS, SMGs were defined as genes which showed classical m6A enrichment around the stop codons. White color means without m6A peak, green color means with m6A peak. **(E)** The m6A topological patterns along the m6A deposited regions for mouse adult tissues, including heart, lung.


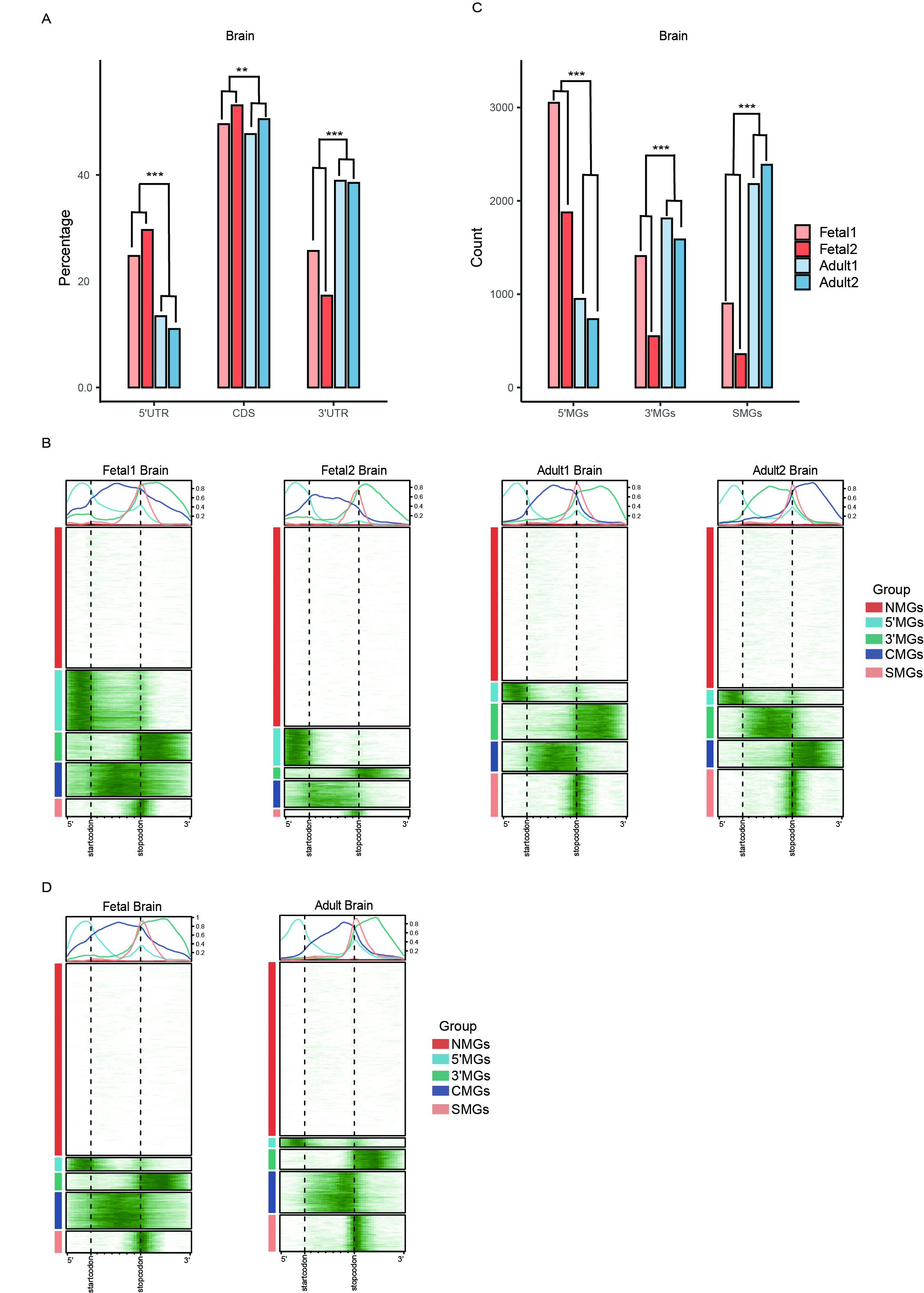


**Supplementary Figure 2. m6A profile in human and mouse brain tissues. (A)** The frequency of m6As located in the 5′ UTRs, CDS and 3′ UTRs among human brain tissues in fetal and adult stages (*z*-test*,* ****P* value < 0.001, ** *P* value < 0.01). Fetal1 means the first dataset of adult tissues and fetal2 means the second datasets, adult1 means the first dataset of adult tissues and adult2 means the second datasets. **(B)** The m6A topological patterns along the m6A deposited regions in human fetal and adult brain tissues. White color means without m6A peak, green color means with m6A peak. **(C)** The frequency of 5′MGs, 3′MGs and SMGs among human brain tissues in fetal and adult stages (*z*-test, ****P* value < 0.001, * *P* value < 0.05, ns: not significantly). **(D)** The m6A topological patterns along the m6A deposited regions in mouse fetal and adult brain tissues.


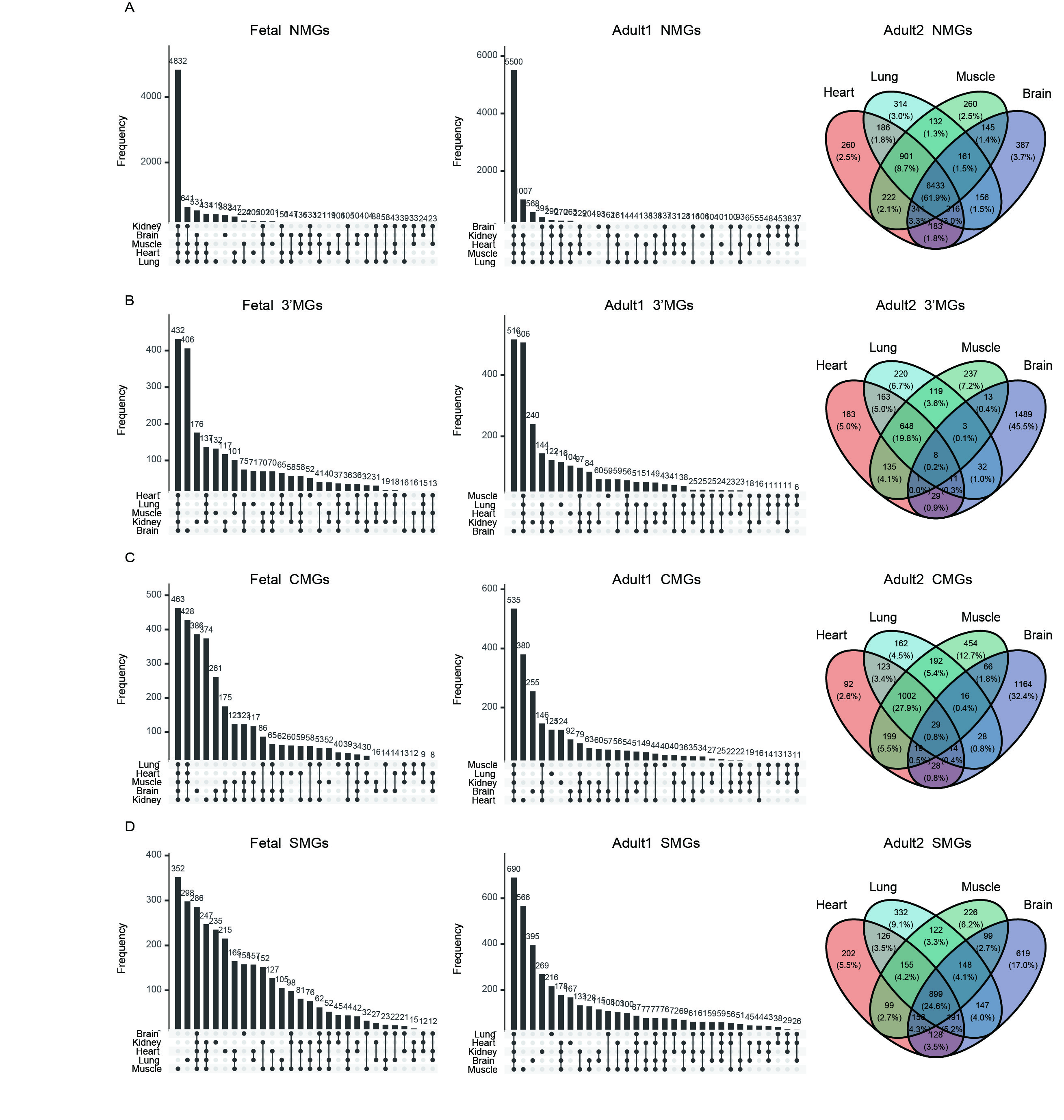


**Supplementary Figure 3. The overlap between each group of the fetal and adult tissues.** The overlap of **(A)** NMGs, **(B)** 3′MGs, **(C)** CMGs, **(D)** SMGs across all analyzed tissues in fetal and adult stages. Adult1 means the first dataset of adult tissues and adult2 means the second datasets.

**
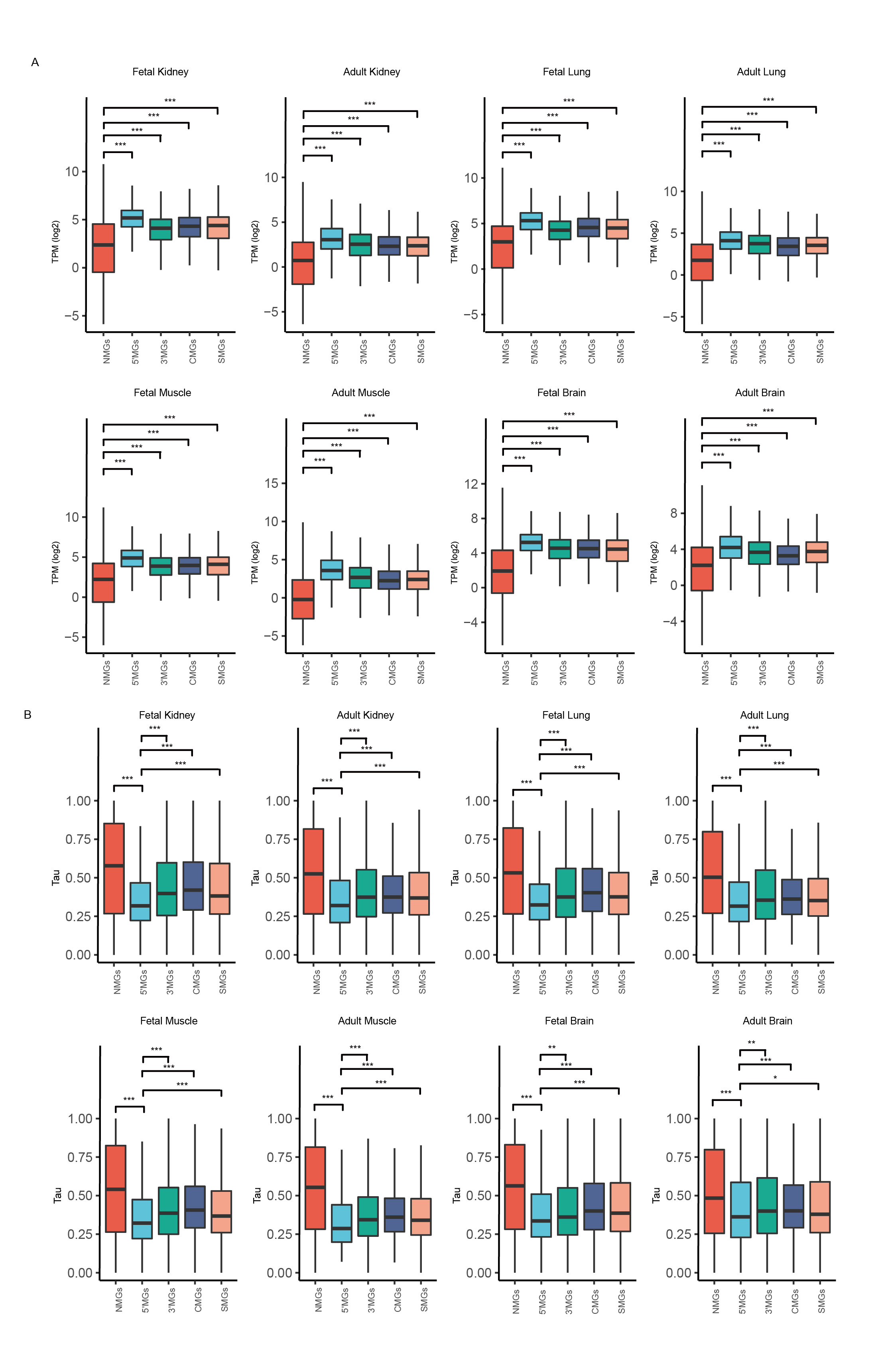
**

**Supplementary Figure 4. Functional characterization of gene groups with different m6A topologies in human tissues.**  **(A)** The expression level (transcripts per kilobase per million mapped reads, TPM) of five group genes across human tissues in fetal and adult stages (ANOVA, ****P* value < 0.001). **(B)** The tissue specificity score tau of five gene groups across human tissues in fetal and adult stages (Kruskal-Wallis rank-sum test, ****P* value < 0.001).

**
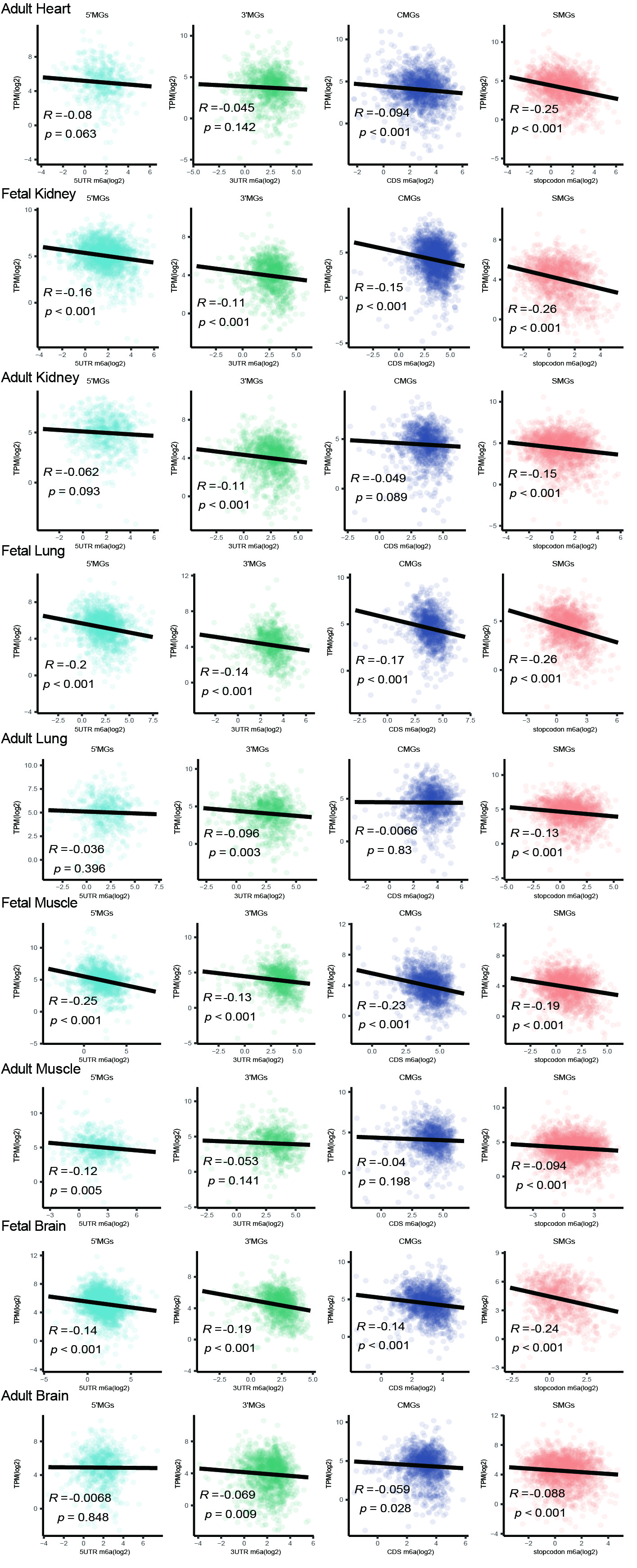
**

**Supplementary Figure 5.**  **Scatter plots showed the correlation between expression level and m6A methylation at different genic regions**.


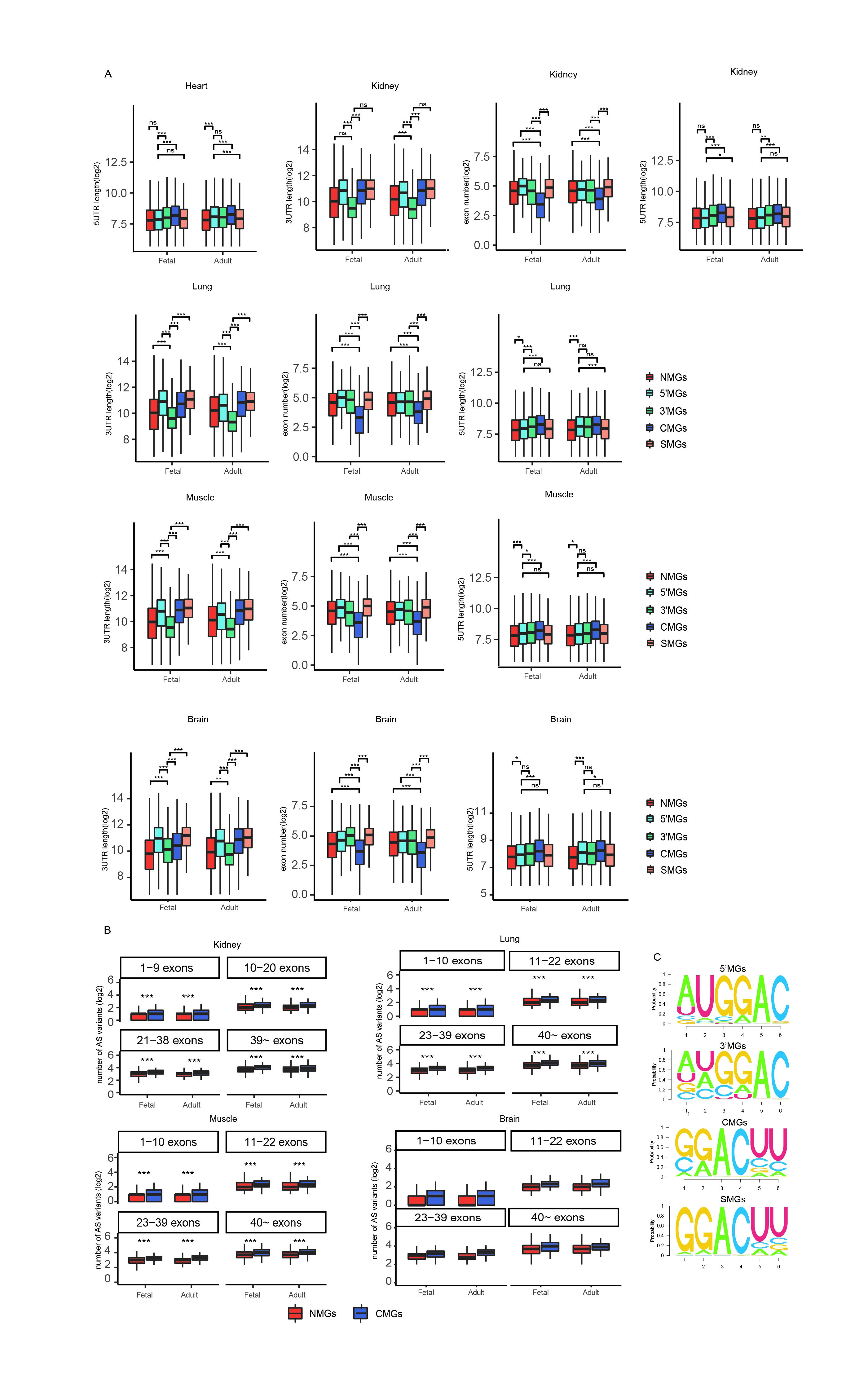


**Supplementary Figure 6. Potential mechanisms associated with different m6A topologies among different gene groups. (A)** The 3′UTRs length, exon number and 5′UTRs length of each gene group among human tissues in fetal and adult stages (Kruskal-Wallis rank-sum test, Wilcoxon rank-sum test, ****P value* < 0.001, ***P* value < 0.01, **P* value < 0.05, ns: not significantly, *P* value > 0.05). **(B)** The number of alternative splicing (AS) variants in NMGs and CMGs with distinct exon numbers across human tissues in fetal and adult stages (Wilcoxon rank-sum test, ****P value* < 0.001). **(C)** The m6A peaks in5’MGs, 3’MGs, CMGs and SMGs all preferred to deposited in the GGACH sub-motif.

**
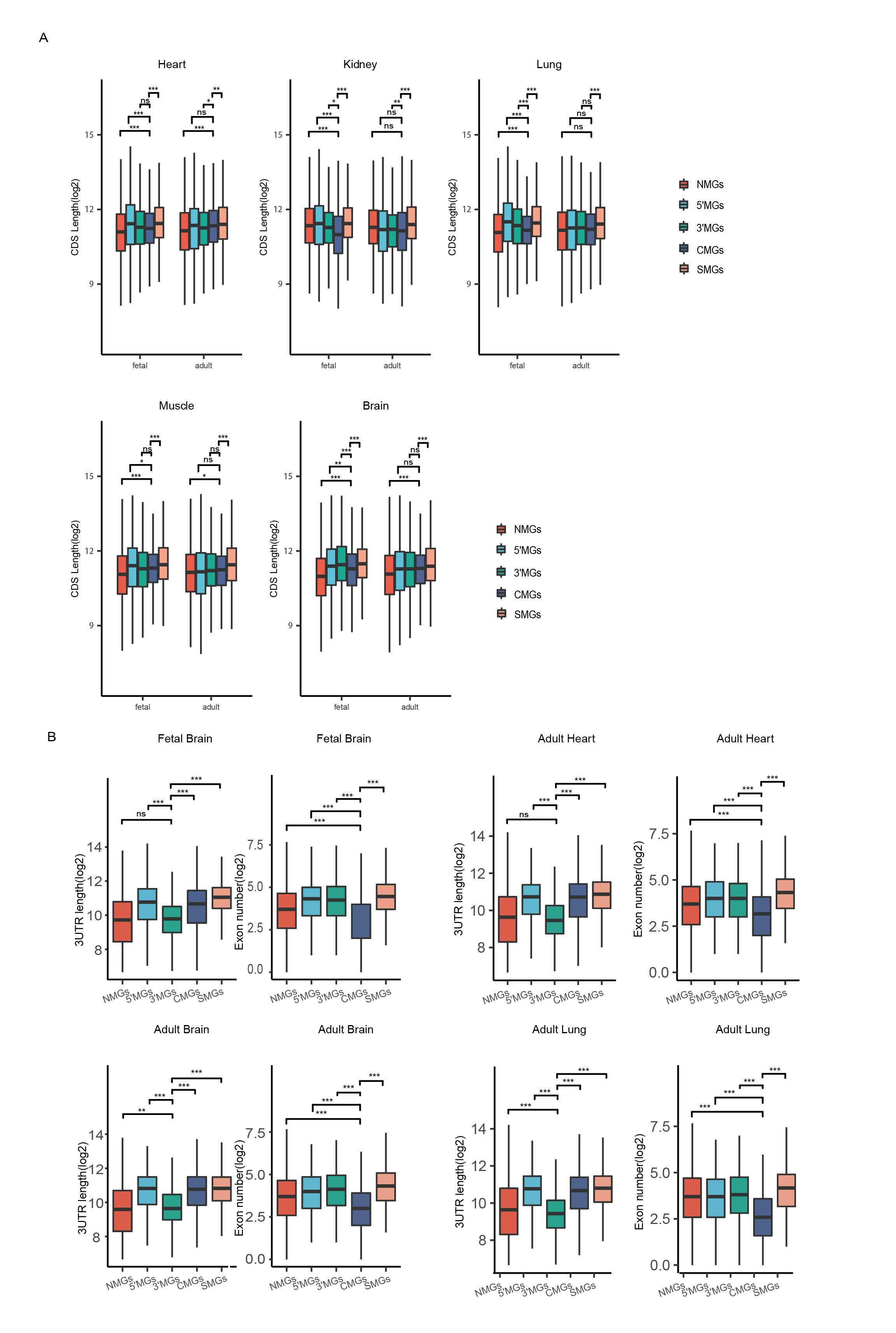
**

**Supplementary Figure 7. Characterization of gene groups with different m6A topologies and topological transition in human and mouse tissues. (A)** The CDS length of each gene group among human tissues in fetal and adult stages (Kruskal-Wallis rank-sum test, ****P value* < 0.001, ***P* value < 0.01, **P* value < 0.05, ns: *P* value > 0.05). **(B)** The 3′UTRs length, exon number of each gene group across mouse tissues (Kruskal-Wallis rank-sum test, ****P* value < 0.001).

**
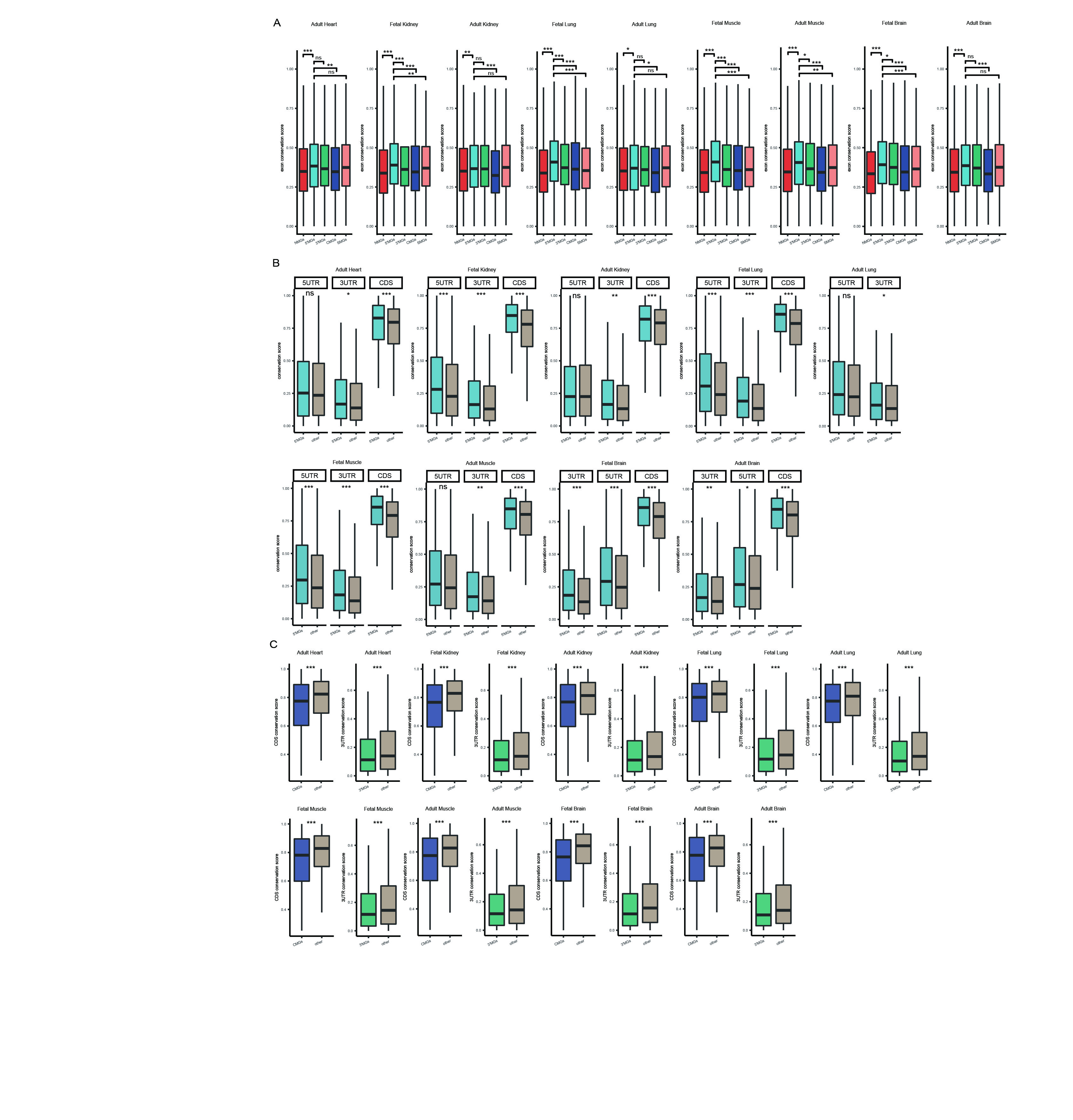
**

**Supplementary Figure 8. Conservation score compared among different gene groups across all tissues.**  **(A)** The exon conservation score of five gene groups across human tissues in fetal and adult stages (Kruskal-Wallis rank-sum test, ****P value* < 0.001). **(B)** The 5′UTRs conservation score, 3′UTRs conservation score, and CDS conservation score among 5′MGs and other group genes with m6A methylation across human tissues in fetal and adult stages (Wilcoxon rank-sum test, ****P value* < 0.001, ***P value* < 0.01, **P value* < 0.05, ns: not significantly, *P* value > 0.05). **(C)**  The CDS conservation score of CMGs and other group genes with m6A methylation; the 3′UTRs conservation score of 3′MGs and other group genes with m6A methylation in human fetal kidney tissue across human tissues in fetal and adult stages (Wilcoxon rank-sum test, ****P value* < 0.001).

**
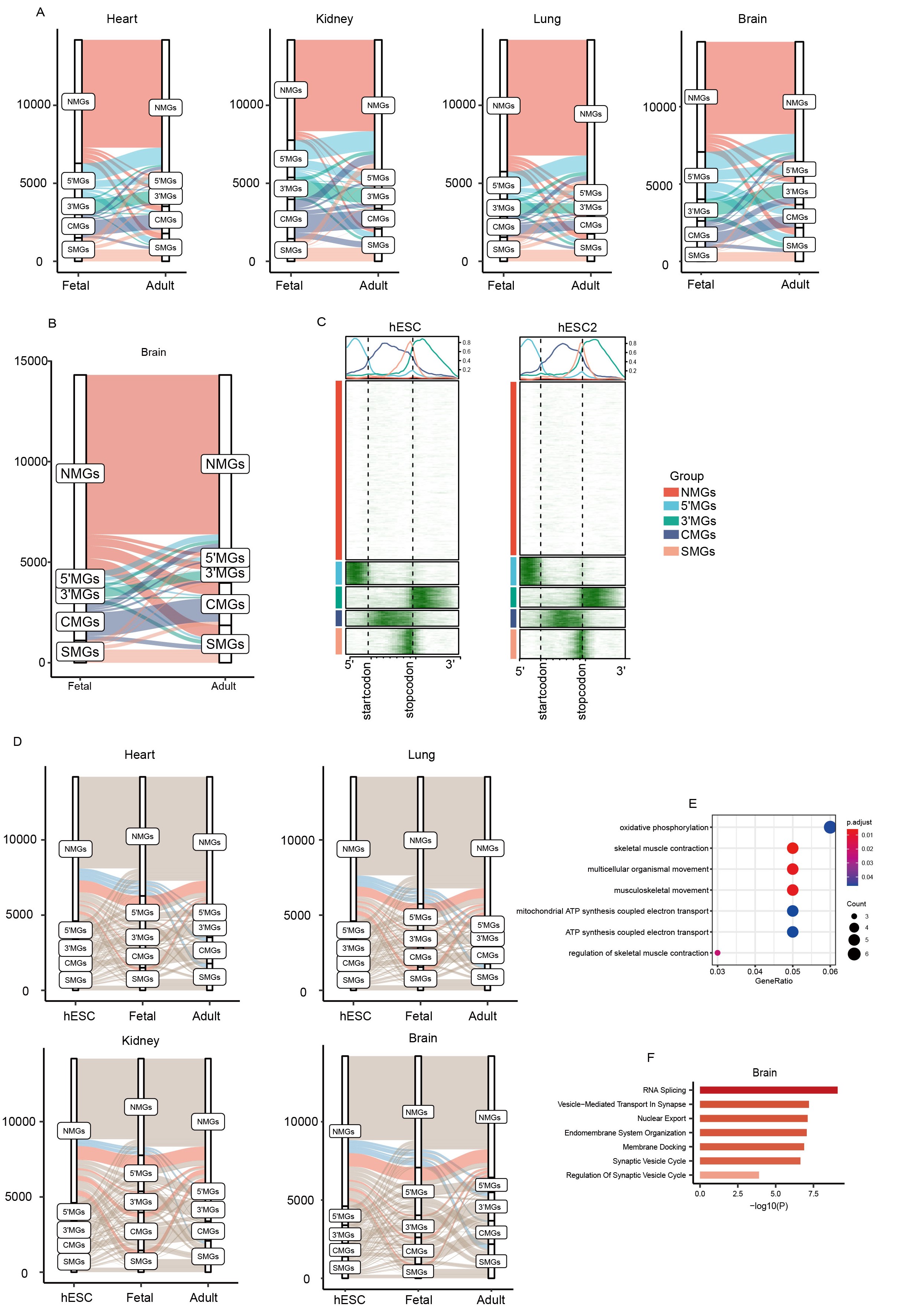
**

**Supplementary Figure 9. Dynamic m6A topological transition during mammalian tissue development which was associated with tissue development.**  **(A)** The m6A topological transition between human tissues in fetal and adult stages. **(B)** The transition between fetal and adult of mouse brain tissue. **(C)** The m6A topological patterns along the m6A deposited regions in two hESC samples (each has three replicates). White color means without m6A peak, green color means with m6A peak. **(D)** The m6A topological transition between three developmental stages across all human tissues. **(E)** Gene ontology enrichment analysis of muscle adult-specific 5′MGs. **(F)** Gene ontology enrichment of brain adult-loss 5′MGs.

**
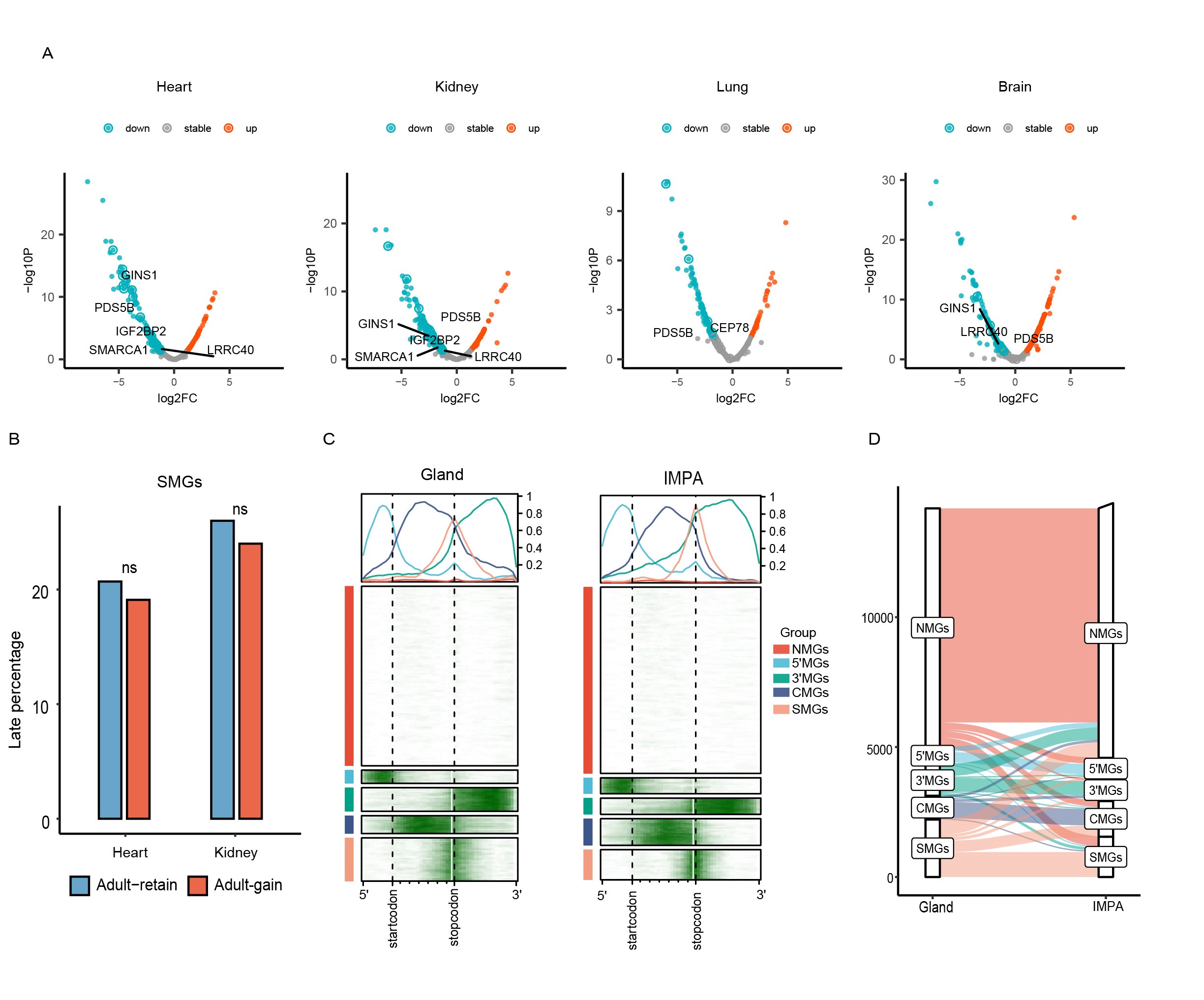
**

**Supplementary Figure 10. m6A topological transition patterns between fetal and adult tissues and normal and tumor tissues.** **(A)** Volcano plot showing the distribution of all differentially expressed adult-loss 5′MGs across all human tissues during tissue development. LogFC means the fold change (log2) of expression level during tissue development. **(B)** The distribution of late genes in adult-gain and adult-retain SMGs across human tissues (*χ*^2^ test, ns: not significantly, *P* value > 0.05). Those with significant increased expression were classified as “late” genes. (**C**) The m6A topological patterns along the m6A deposited regions in human normal gland and invasive malignant pleomorphic adenoma (IMPA). White color means without m6A peak, green color means with m6A peak. **(D)** The m6A topological transition between normal gland and IMPA.
