# Supplementary figures and images for: m6A Topological Transition Coupled to Developmental Regulation of Gene Expression During Mammalian Tissue Development
Source: Front Cell Dev Biol. 2022 Jul 5;10:916423. doi: 10.3389/fcell.2022.916423 (PMC9294180; doi:10.3389/fcell.2022.916423)

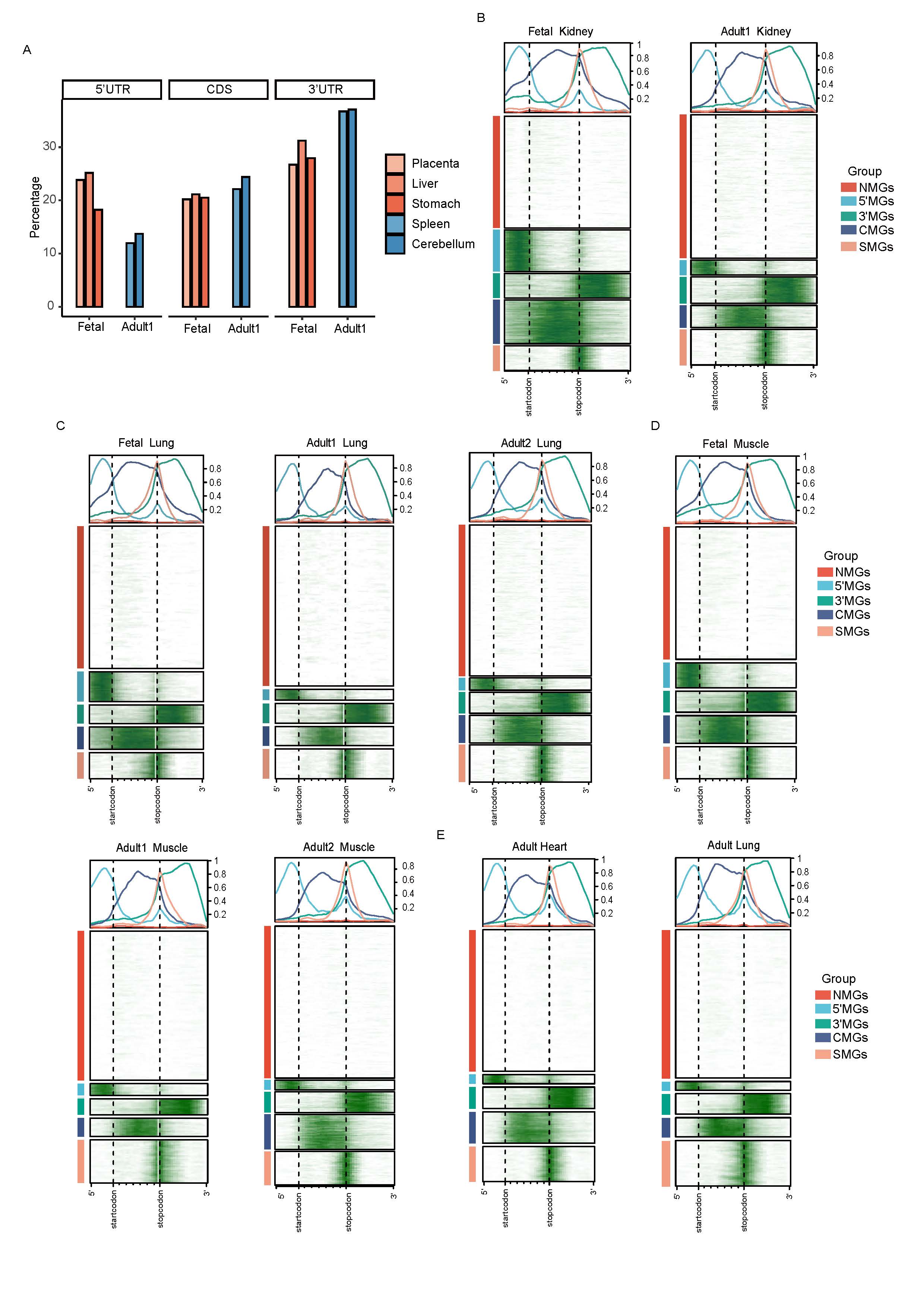

Supplement: Supplementary file 1 [file DataSheet1.zip › Additional Files/Figure S1.jpg]

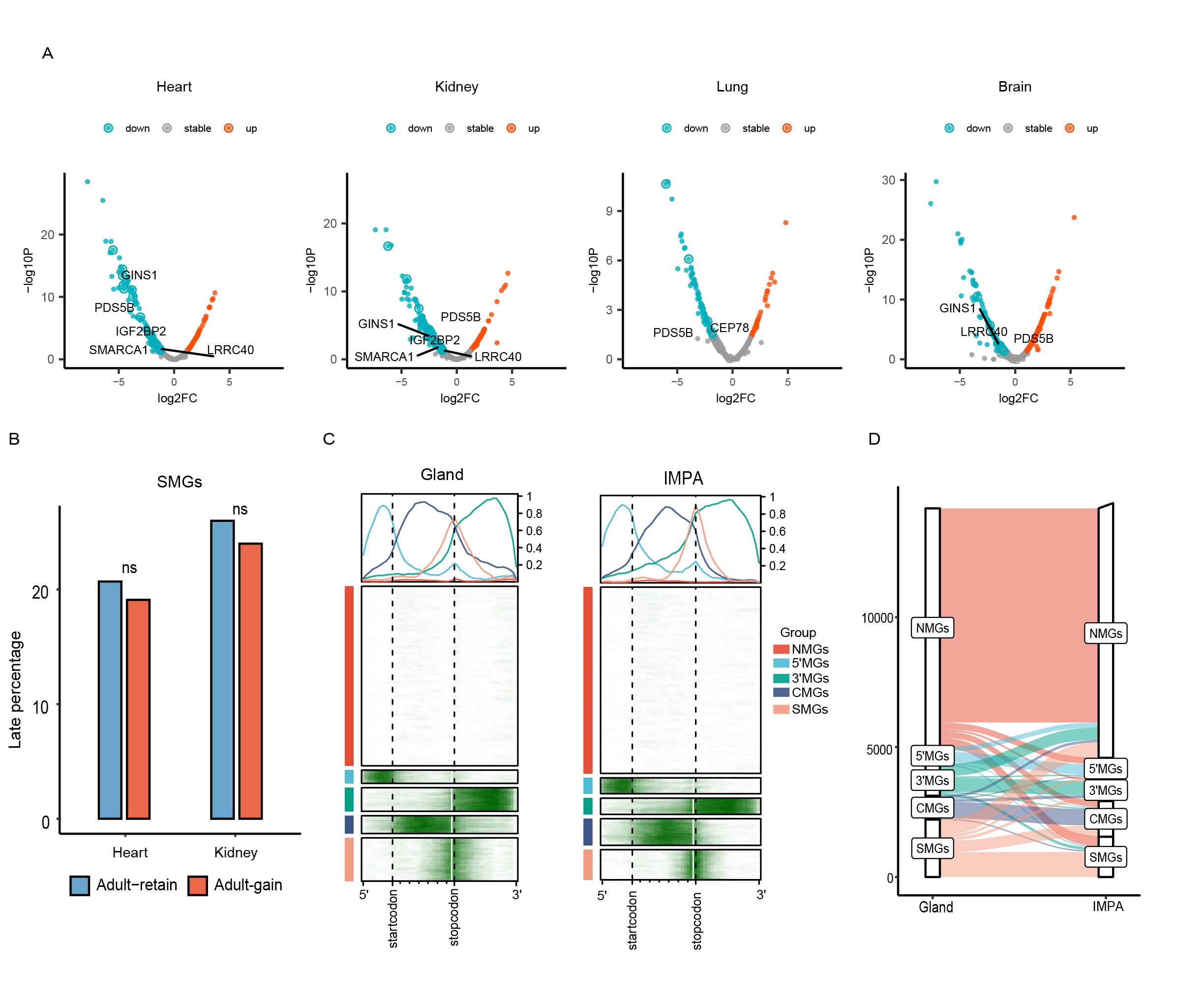

Supplement: Supplementary file 1 [file DataSheet1.zip › Additional Files/Figure S10.jpg]

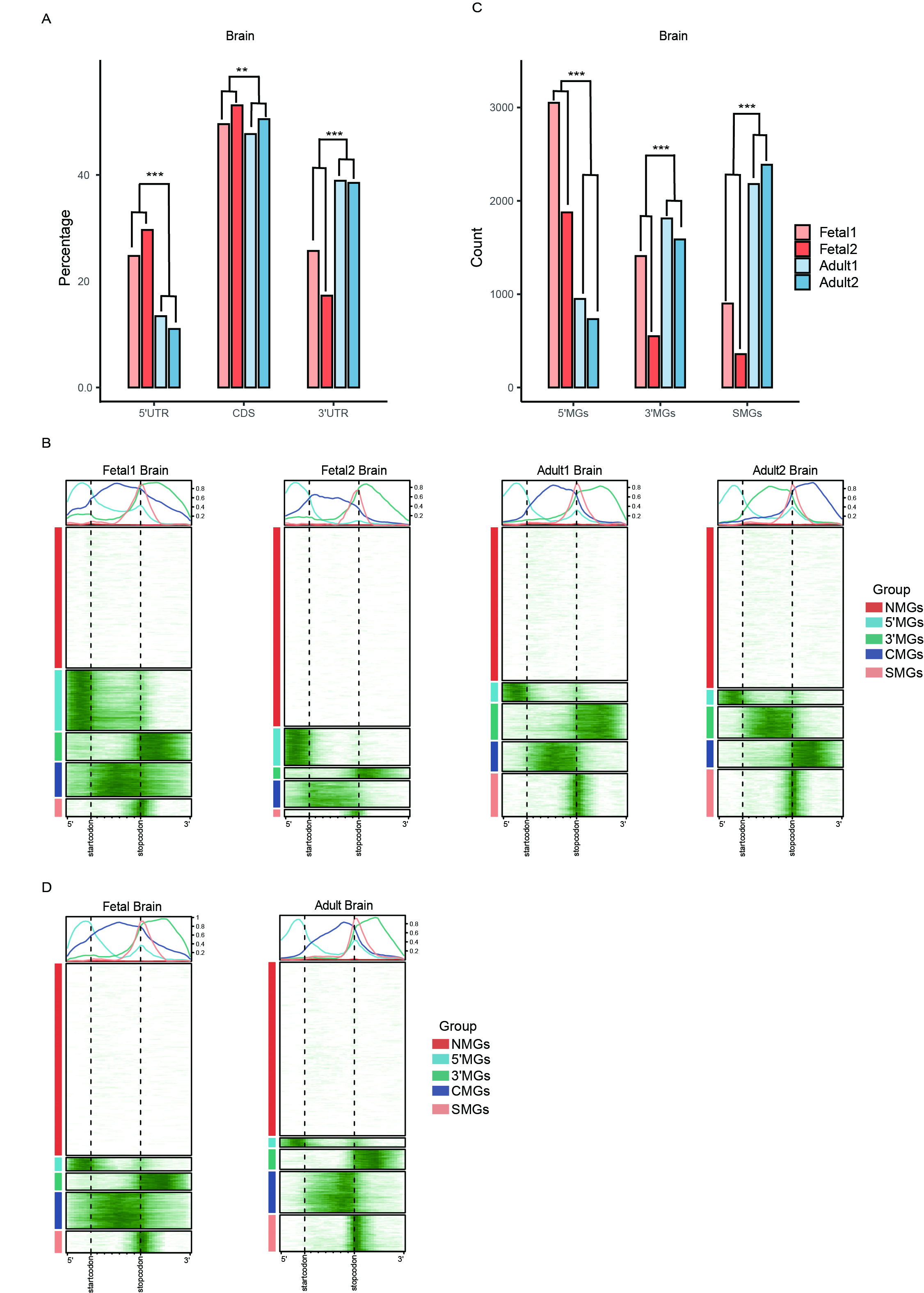

Supplement: Supplementary file 1 [file DataSheet1.zip › Additional Files/Figure S2.jpg]

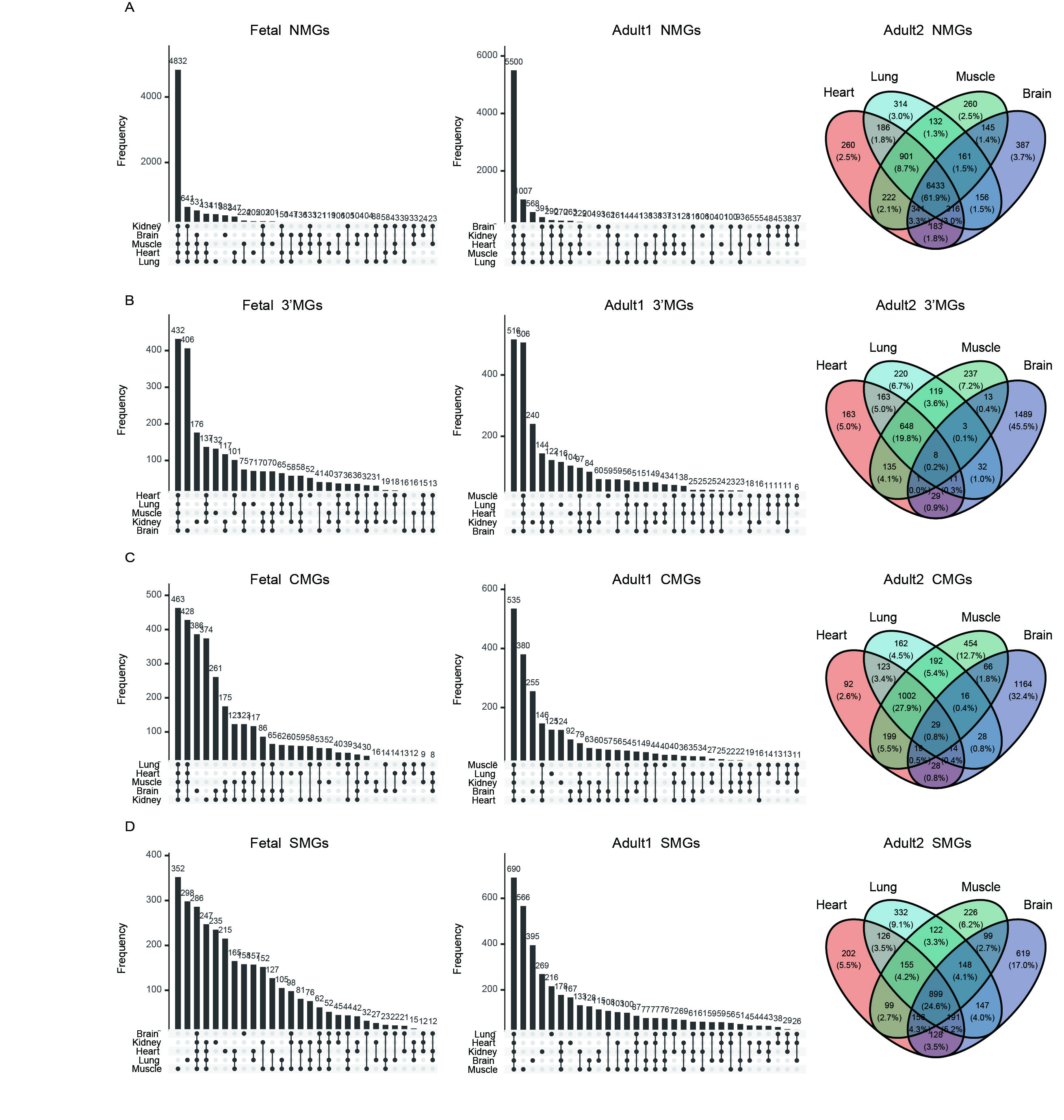

Supplement: Supplementary file 1 [file DataSheet1.zip › Additional Files/Figure S3.jpg]

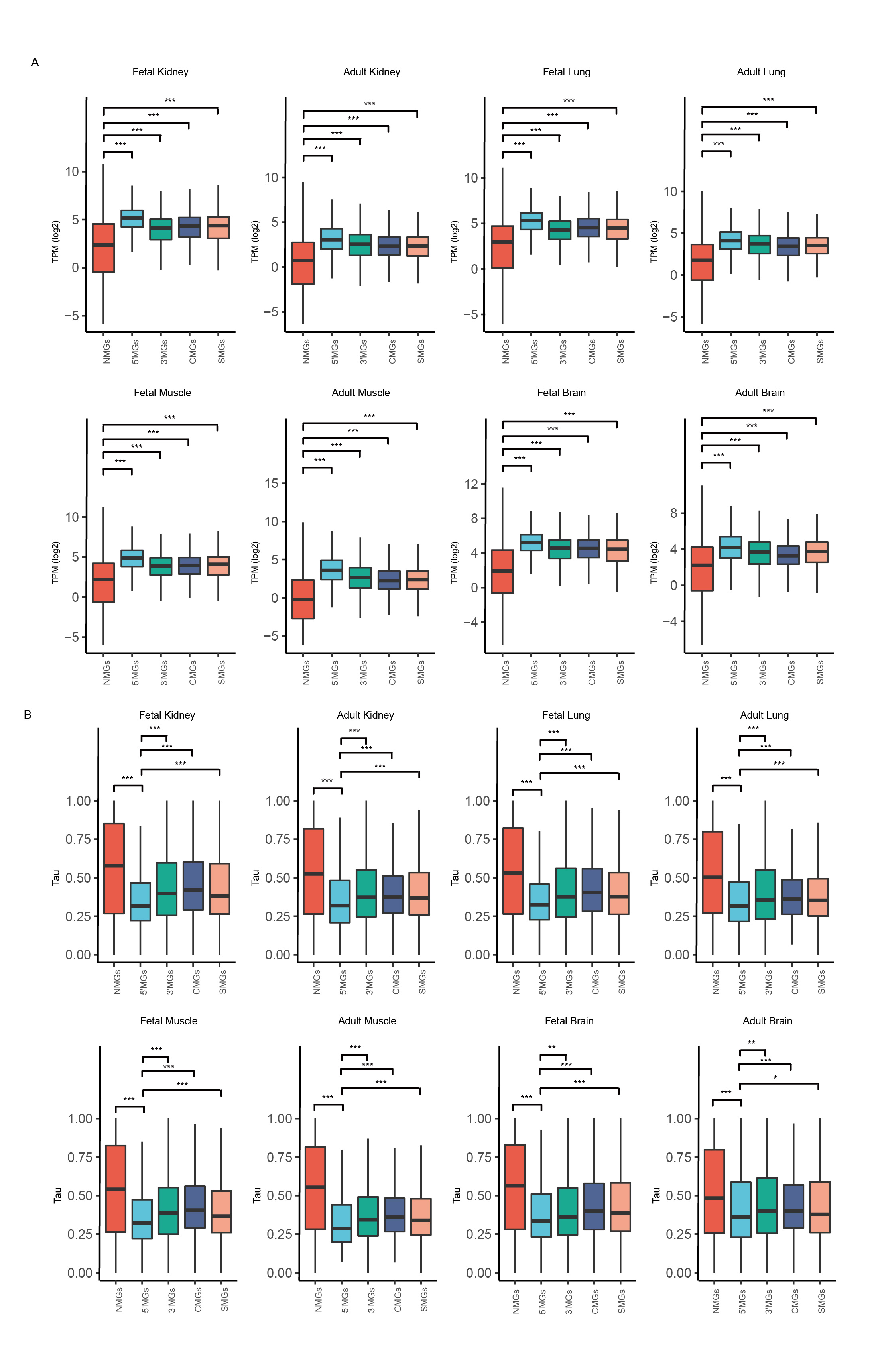

Supplement: Supplementary file 1 [file DataSheet1.zip › Additional Files/Figure S4.jpg]

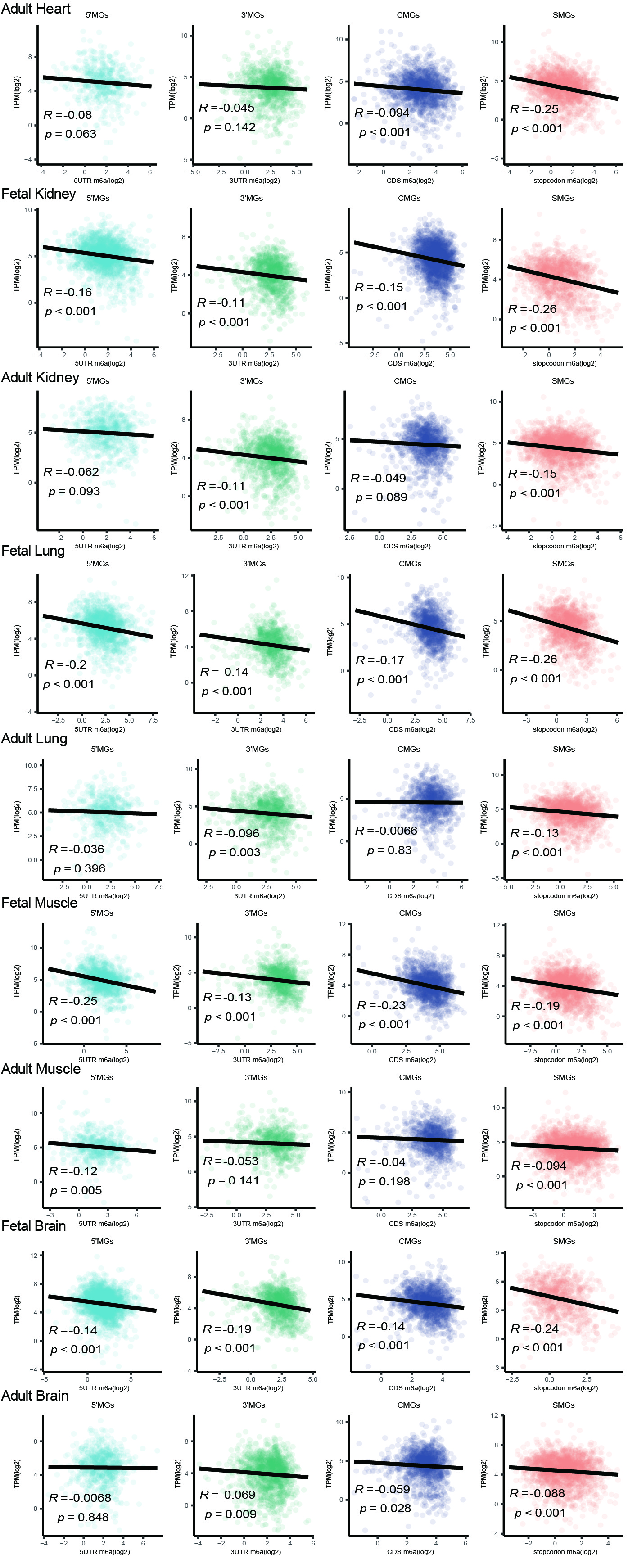

Supplement: Supplementary file 1 [file DataSheet1.zip › Additional Files/Figure S5.jpg]

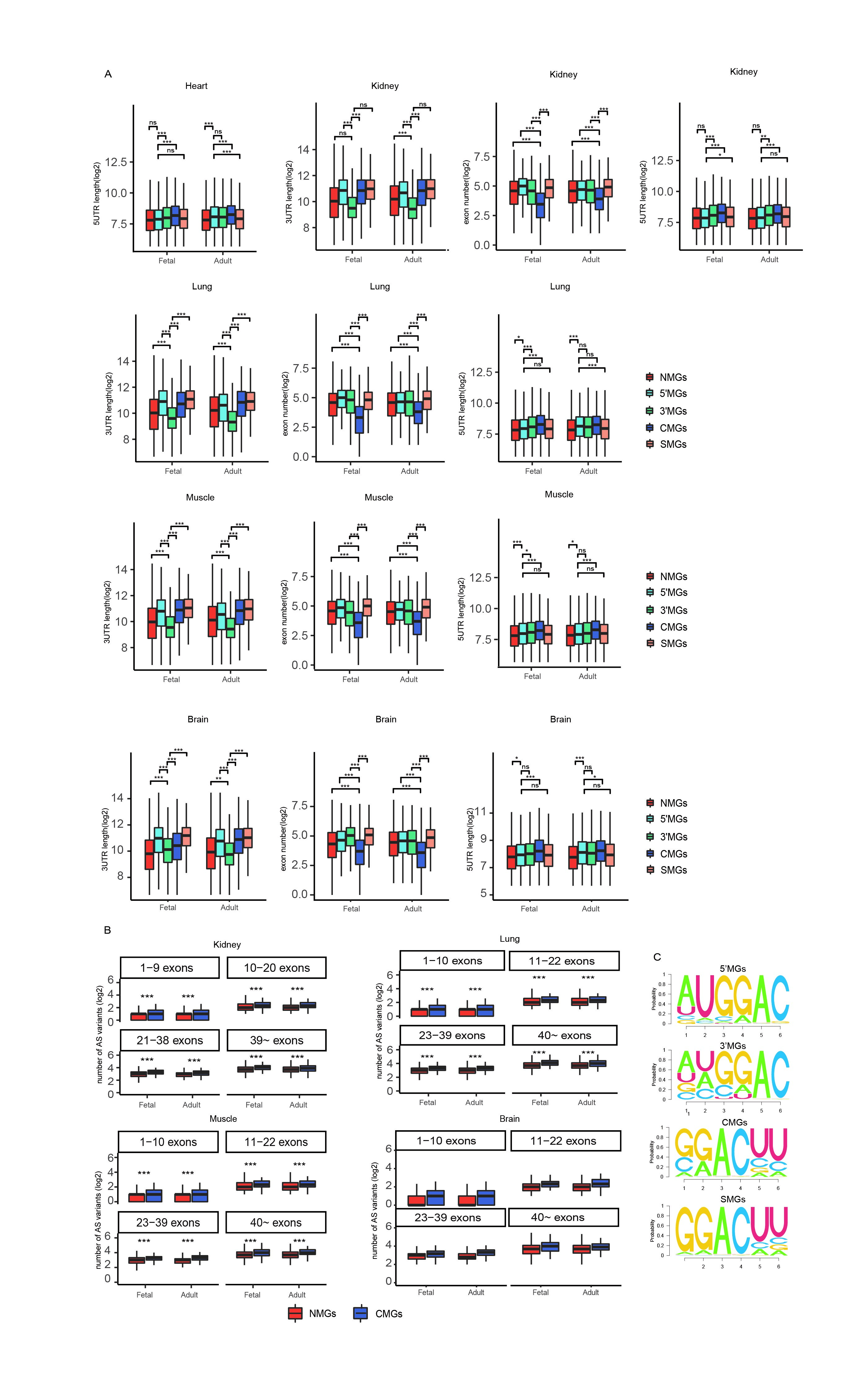

Supplement: Supplementary file 1 [file DataSheet1.zip › Additional Files/Figure S6.jpg]

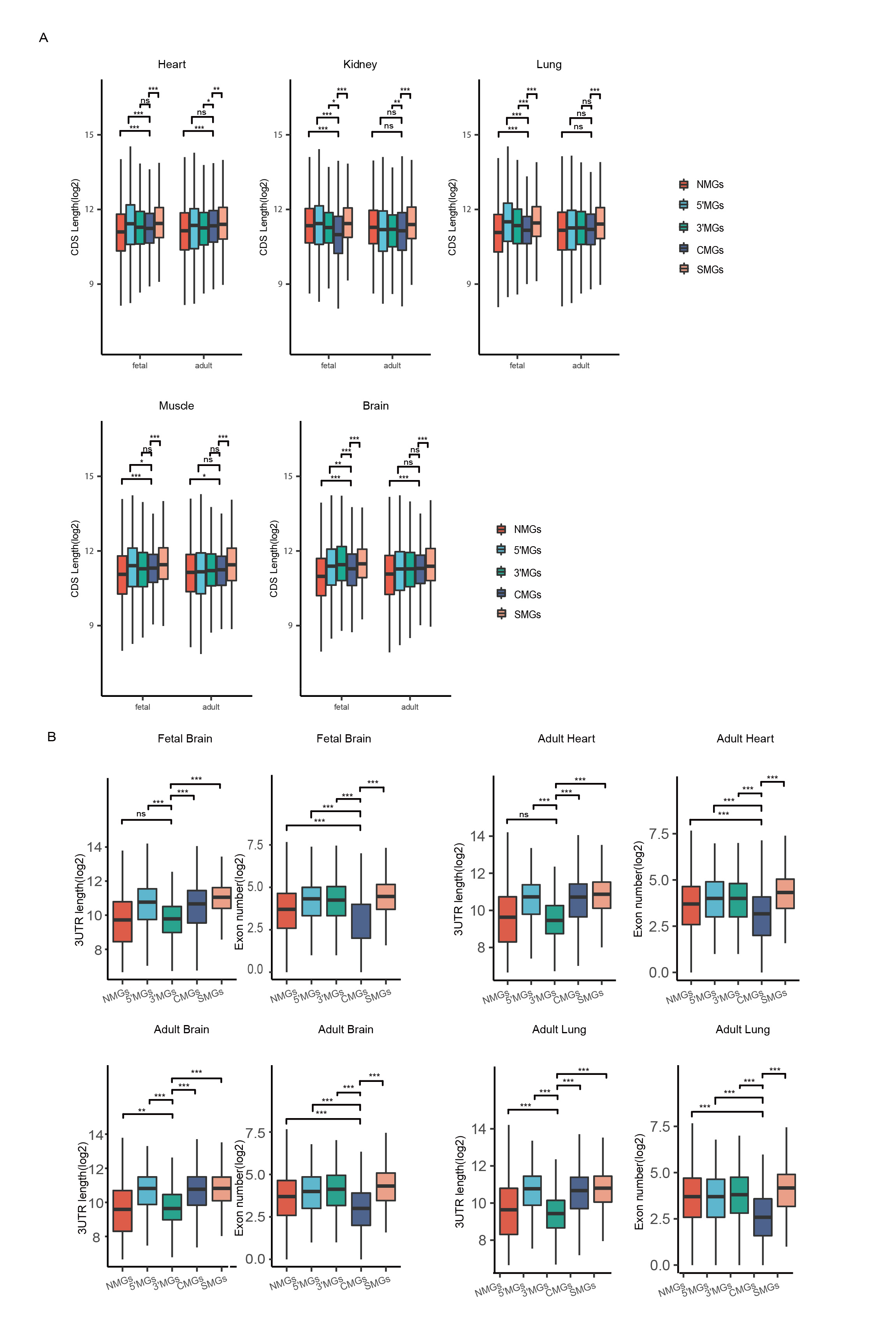

Supplement: Supplementary file 1 [file DataSheet1.zip › Additional Files/Figure S7.jpg]

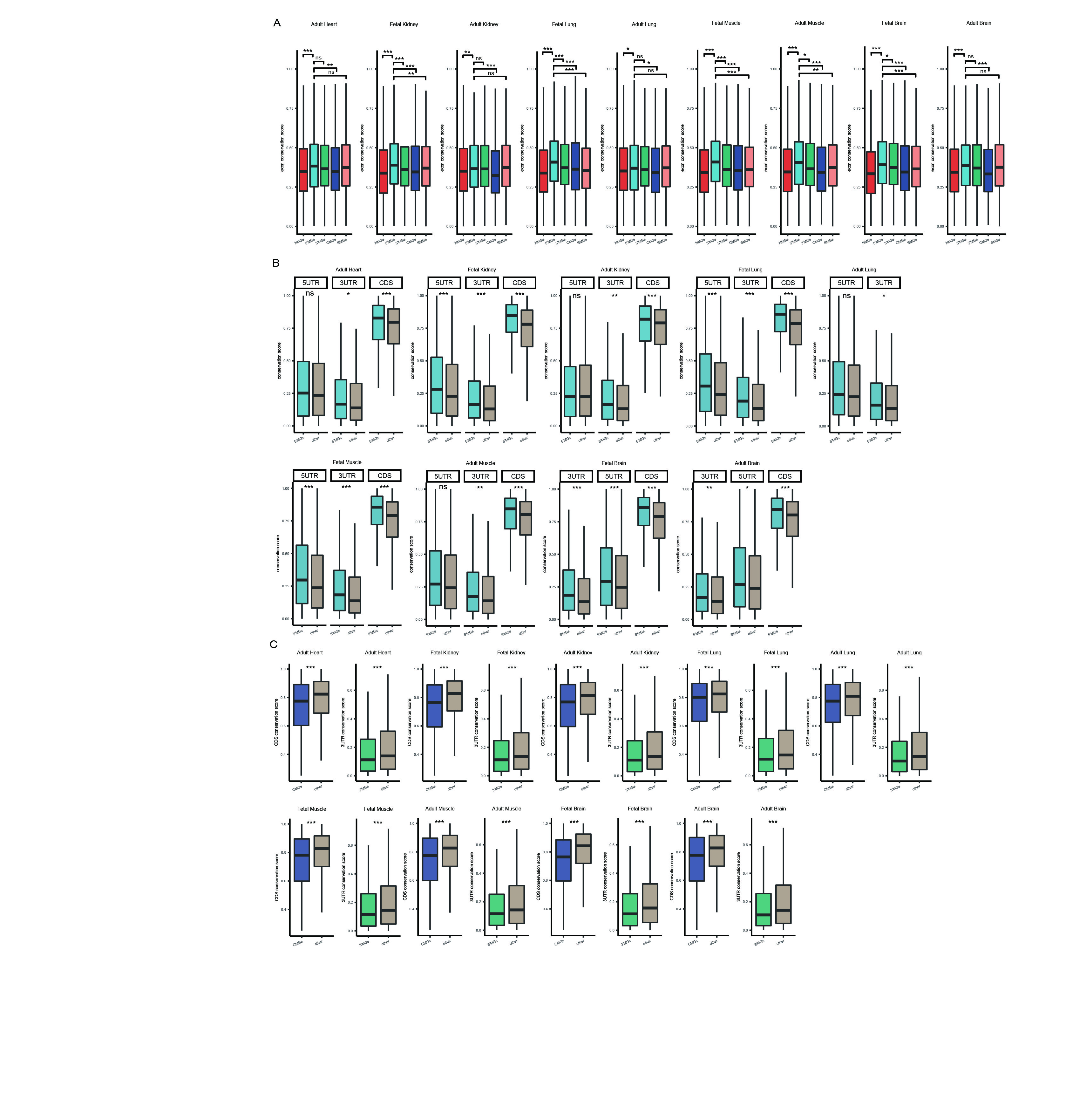

Supplement: Supplementary file 1 [file DataSheet1.zip › Additional Files/Figure S8.jpg]

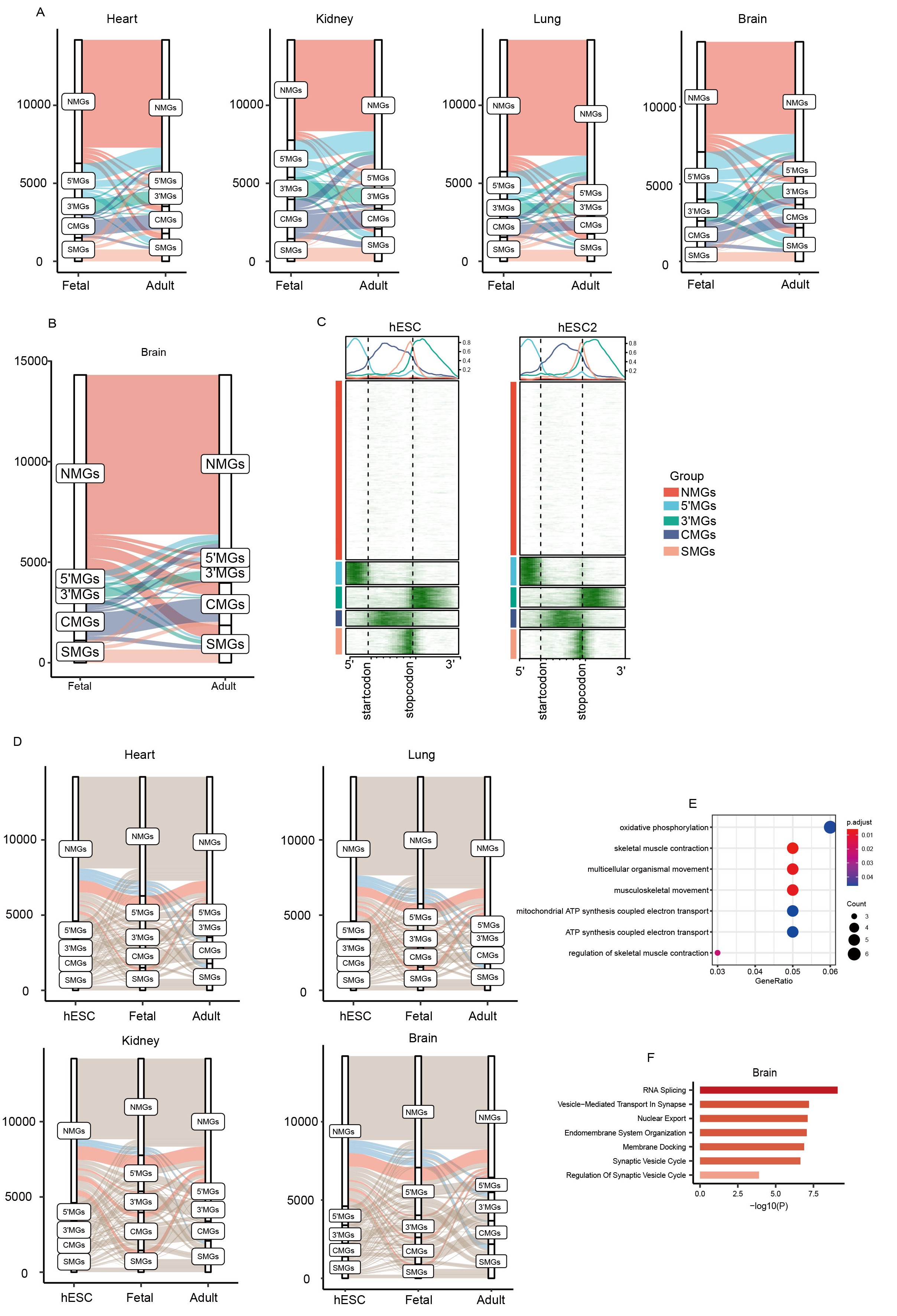

Supplement: Supplementary file 1 [file DataSheet1.zip › Additional Files/Figure S9.jpg]
